# Disulfide bond disrupting agents activate the unfolded protein response in EGFR- and HER2-positive breast tumor cells

**DOI:** 10.18632/oncotarget.15952

**Published:** 2017-03-07

**Authors:** Renan B. Ferreira, Mengxiong Wang, Mary E. Law, Bradley J. Davis, Ashton N. Bartley, Paul J. Higgins, Michael S. Kilberg, Katherine E. Santostefano, Naohiro Terada, Coy D. Heldermon, Ronald K. Castellano, Brian K. Law

**Affiliations:** ^1^ Department of Chemistry, University of Florida, Gainesville, FL 32611, USA; ^2^ Department of Pharmacology & Therapeutics, University of Florida, Gainesville, FL 32610, USA; ^3^ UF-Health Cancer Center, University of Florida, Gainesville, FL 32610, USA; ^4^ Center for Cell Biology and Cancer Research, Albany Medical College, Albany, NY 12208, USA; ^5^ Department of Biochemistry, University of Florida, Gainesville, FL, 32610, USA; ^6^ Department of Pathology, Immunology, and Laboratory Medicine, Center for Cellular Reprogramming, University of Florida College of Medicine, Gainesville, FL 32610, USA; ^7^ Department of Medicine, University of Florida, Gainesville, FL, 32610, USA

**Keywords:** HER2, EGFR, UPR, Akt, breast cancer

## Abstract

Many breast cancer deaths result from tumors acquiring resistance to available therapies. Thus, new therapeutic agents are needed for targeting drug-resistant breast cancers. Drug-refractory breast cancers include HER2+ tumors that have acquired resistance to HER2-targeted antibodies and kinase inhibitors, and “Triple-Negative” Breast Cancers (TNBCs) that lack the therapeutic targets Estrogen Receptor, Progesterone Receptor, and HER2. A significant fraction of TNBCs overexpress the HER2 family member Epidermal Growth Factor Receptor (EGFR). Thus agents that selectively kill EGFR+ and HER2+ tumors would provide new options for breast cancer therapy. We previously identified a class of compounds we termed Disulfide bond Disrupting Agents (DDAs) that selectively kill EGFR+ and HER2+ breast cancer cells *in vitro* and blocked the growth of HER2+ breast tumors in an animal model. DDA-dependent cytotoxicity was found to correlate with downregulation of HER1-3 and Akt dephosphorylation. Here we demonstrate that DDAs activate the Unfolded Protein Response (UPR) and that this plays a role in their ability to kill EGFR+ and HER2+ cancer cells. The use of breast cancer cell lines ectopically expressing EGFR or HER2 and pharmacological probes of UPR revealed all three DDA responses: HER1-3 downregulation, Akt dephosphorylation, and UPR activation, contribute to DDA-mediated cytotoxicity. Significantly, EGFR overexpression potentiates each of these responses. Combination studies with DDAs suggest that they may be complementary with EGFR/HER2-specific receptor tyrosine kinase inhibitors and mTORC1 inhibitors to overcome drug resistance.

## INTRODUCTION

The principle of “oncogene addiction” [1–3] posits that particular cancers developed and continue to grow and survive only by virtue of their initiating oncogene. This principle has guided much of targeted cancer therapy and has led to treatments for breast tumors that overexpress the Human Epidermal Growth Factor Receptor-2 (HER2) receptor tyrosine kinase. These drugs include the HER2 targeted monoclonal antibodies Trastuzumab [4–7] and Pertuzumab [8–10] and the HER2/Epidermal Growth Factor Receptor (EGFR/HER1) tyrosine kinase inhibitor Lapatinib [11–14]. HER2 targeted drugs have significantly improved the survival of patients with HER2+ breast tumors, but tumor resistance to these agents remains a significant problem [15–18]. Thus, in addition to monoclonal antibodies and tyrosine kinase inhibitors, other therapeutic strategies are needed to effectively treat patients whose tumors have acquired resistance to HER2/EGFR targeted agents. A significant source of drug resistance is the functional redundancy among EGFR, HER2, and HER3, whereby inactivation of one family member may be compensated for by another family member [19–22]. Thus, drugs that act on features common to HER1-3 could make a significant impact on tumors driven by the HER2 or EGFR that have become refractory to available targeted therapies.

Cancer is associated with dysregulation of protein synthesis and protein folding, referred to as defective proteostasis [23, 24]. HER2-positive breast cancers are addicted to Endoplasmic Reticulum-Associated protein Destruction (ERAD) [25]. This may result from the reliance of these cancers on HER-family proteins and the fact that HER-family members EGFR, HER2, HER3, and HER4 share conserved extracellular cysteine-rich repeats that form numerous disulfide bonds that on overexpression may present a burden to the protein folding machinery.

Compounds that disrupt the folding of HER-family proteins may provide an effective means to target cancers that are addicted to these receptors and could complement other classes of HER2-specific agents. Management of patients with HER2+ breast cancers involves combining these HER2-targeted agents with conventional chemotherapy drugs. Further, primary and acquired resistance to HER2-specific therapy is a frequent occurrence that leaves patients with few options. Hence new agents that cooperate with existing HER2-specific therapeutics could replace cytotoxic chemotherapy drugs, and might be useful for overcoming resistance by simultaneously downregulating HER1-3.

EGFR also contributes to the development and progression of breast cancers [26, 27] and is overexpressed in approximately 50% of the Triple-Negative Breast Cancers (TNBCs) that lack Estrogen Receptor (ER), Progesterone Receptor (PR), and HER2 [28]. Subsets of lung cancers and glioblastomas harbor activated point mutants or splice variants of EGFR, but such alterations are rare in breast cancer where EGFR is activated primarily by overexpression of the wild type protein [28]. Thus agents that selectively block the folding of HER1-3 could have a major impact on the treatment of breast cancer.

The molecular chaperone Hsp90 facilitates the folding of a number of oncoproteins including HER2 [29, 30]. The Hsp90 inhibitor Geldanamycin and its analogs are under investigation as anticancer agents [31]. Progress in moving Geldanamycin analogs toward clinical application has been slowed by problems relating to drug toxicity and solubility [32]. We recently described a class of agents termed Disulfide bond Disrupting Agents (DDAs) that are capable of breaking disulfide bonds in solution, and when applied to breast cancer cells downregulate HER family members EGFR, HER2, and HER3 in parallel, and inactivate Akt [33]. These agents have the potential to ablate drug resistance by overcoming the functional redundancy among HER1-3 and act downstream at the level of Akt to abrogate drug resistance mediated through upstream activation of Phosphatidylinositol 3-Kinase (PI3K). Here we show that in addition to these useful properties, DDAs also activate the Unfolded Protein Response (UPR). Importantly, DDA-induced UPR is potentiated by overexpression of EGFR or HER2, providing a partial explanation as to how DDAs can effectively kill cancer cells without harming normal tissues. DDAs are chemically and mechanistically distinct from other classes of anticancer agents and selectively exacerbate the ER stress caused by the aberrantly high expression of EGFR and HER2 that occurs in breast cancers.

## RESULTS

### DDAs induce ER stress

**Figure 1:**
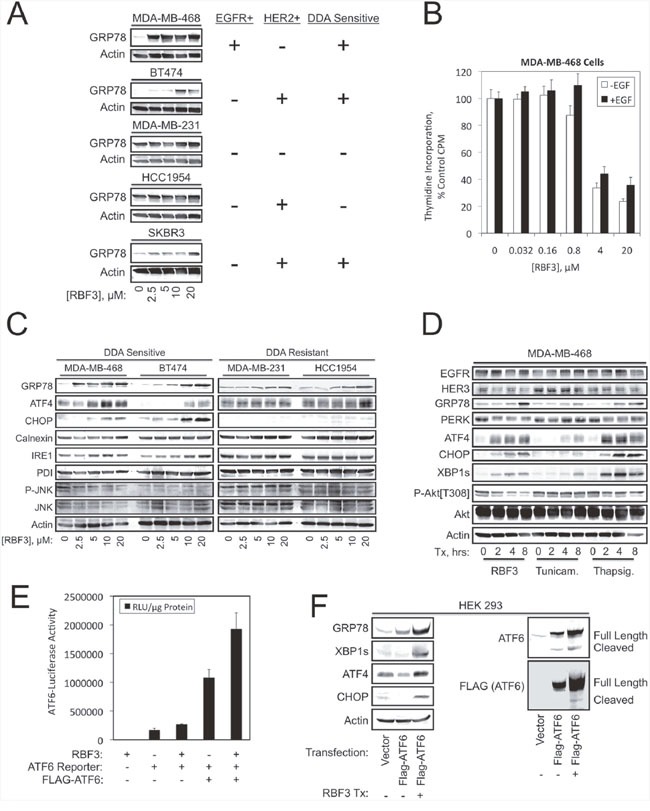
DDA responsiveness parallels activation of the unfolded protein response **(A)** The specified cell lines were treated with the indicated RBF3 concentrations for 24 hours and analyzed for the ER stress marker GRP78 by immunoblot (left panel). The right panel indicates the status of EGFR or HER2 expression and DDA sensitivity. **(B)** Proliferation of MDA-MB-468 cells treated for 24 hours with the indicated concentrations of RBF3 in the presence or absence of 20 ng/ml EGF as assessed by tritiated thymidine incorporation. Results are presented as the mean ± standard deviation of triplicate determinations. **(C)** DDA sensitive or resistant cell lines were treated for 24 hours with increasing concentrations of RBF3 and extracts were analyzed by immunoblot for markers related to ER stress. **(D)** The time course of RBF3 responses in MDA-MB-468 cells was compared with that of the ER stress inducers tunicamycin (500 ng/ml) and thapsigargin (400 nM) by immunoblot analysis. **(E)** Luciferase reporter assays measuring the impact of ectopically expressed ATF6 and 20 μM RBF3 on the activity of an ATF6-responsive promoter construct. Results are normalized to micrograms of protein extract assayed, and are presented as the mean ± standard deviation of triplicate determinations. **(F)** Extracts from HEK 293 cells transiently transfected as indicated and treated with or without 20 μM RBF3 for 24 hours were analyzed by immunoblot.

Since disulfide bond formation occurs in the Endoplasmic Reticulum (ER), and HER2+ breast cancers are particularly sensitive to DDAs [33] and ER stress/ERAD [25], we examined whether DDAs (such as RBF3) activate the Unfolded Protein Response (UPR). In the DDA-sensitive MDA-MB-468, BT474, and SKBR3 lines, DDAs activated ER stress as indicated by GRP78 upregulation (Figure 1A). The DDA-resistant MDA-MB-231 and HCC1954 lines exhibited high basal GRP78 expression, suggesting that they have adapted to persistent ER stress. DDAs upregulated GRP78 at the lowest concentrations tested, 2.5 μM, in the MDA-MB-468 line. Suppression of MDA-MB-468 cell proliferation commenced between 0.8 and 4 μM, suggesting that inhibition of cell division and activation of ER stress occur over a similar concentration range (Figure 1B). The ER stress response is mediated by the upstream sensors PERK, IRE1, and ATF6. PERK-dependent activation of an ATF4-CHOP transcriptional axis contributes to cell death in response to un-resolvable ER stress [34, 35]. DDA-sensitive cell lines exhibited upregulation of ATF4 and CHOP in a concentration-dependent manner, while the resistant cell lines expressed high basal ATF4 levels and lacked CHOP expression (Figure 1C). Although the IRE1-Jun kinase axis was previously implicated in ER stress-mediated cell death [36], DDAs did not alter activating Jun kinase (JNK) phosphorylation in any of the cell lines. Comparison of RBF3 with the ER stress inducers tunicamycin and thapsigargin showed that RBF3 elicited ER stress comparably, but was more effective in suppressing Akt phosphorylation (Figure 1D). The observation that RBF3 upregulates XBP1s demonstrates that RBF3 activates the arms of the ER stress response involving the IRE1-XBP1s and PERK-ATF4-CHOP cassettes. Transcriptional reporter assays in HEK293 cells were performed to evaluate whether DDAs activate the third ER stress sensor, ATF6. RBF3 did not significantly stimulate basal ATF6-luciferase activity, but potentiated the transcriptional activity of ectopically expressed ATF6 (Figure 1E). Immunoblot analysis of HEK293 cell extracts demonstrated that overexpression of ATF6 increased endogenous GRP78 expression, but did not increase XBP1s, ATF4, or CHOP levels. RBF3 combined with ATF6 overexpression robustly upregulated GRP78 and increased XBP1s, ATF4, and CHOP levels (Figure 1F). ATF6 activation involves cleavage to release its cytoplasmic domain, which travels to the nucleus to regulate transcription. RBF3 increased the expression of exogenous ATF6, and resulted in higher levels of the cleaved, transcriptionally active form of ATF6. The results in Figure 1 show that DDAs activate all three branches of UPR.

### Ongoing protein synthesis is required for DDA induction of UPR

**Figure 2:**
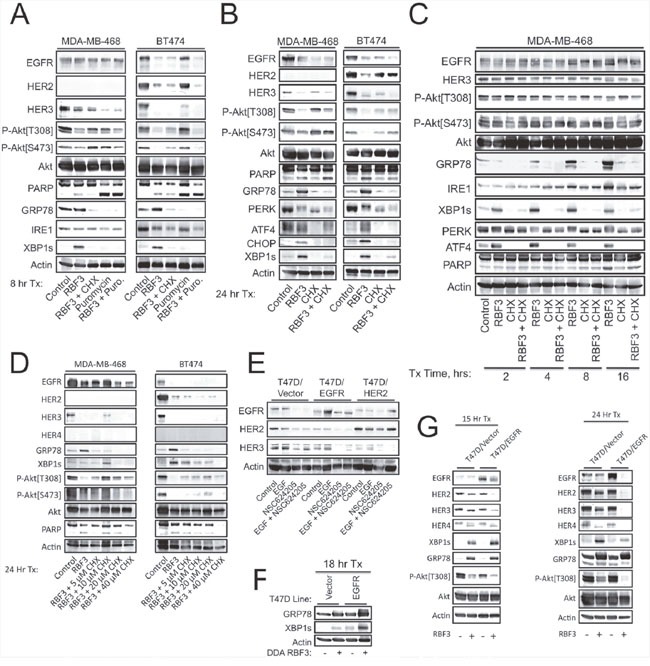
Ongoing protein synthesis is required for DDA induction of UPR, and elevated DDA sensitivity due to forced EGFR or HER3 overexpression correlates with enhanced HER3 downregulation and increased ER stress **(A)** MDA-MB-468 or BT474 cells were treated for 8 hours with 20 μM RBF3, 20 μM Cycloheximide (CHX), or 5 μg/ml Puromycin either alone or in the indicated combinations and cell extracts were analyzed by immunoblot. **(B)** MDA-MB-468 or BT474 cells were treated for 24 hours with 20 μM RBF3 or 20 μM CHX in the indicated combinations and cell extracts were analyzed by immunoblot. **(C)** MDA-MB-468 cells were treated with RBF3 and/or CHX as indicated for 2, 4, 8, or 16 hours and cell extracts were prepared and analyzed by immunoblot. **(D)** MDA-MB-468 or BT474 cells were treated for 24 hours with 20 μM RBF3 combined with increasing concentrations of CHX and cell extracts were analyzed by immunoblot. **(E)** The indicated T47D stable cell lines were treated for 24 hours with 20 ng/ml EGF, 20 μM NSC624205, or EGF + NSC624205 and cell extracts were analyzed by immunoblot. **(F)** Vector control or EGFR overexpressing T47D cells were treated with 20 μM RBF3 for 18 hours and cell extracts were analyzed by immunoblot. **(G)** Vector control or EGFR overexpressing T47D cells were treated with vehicle or 20 μM RBF3 for 15 (left panel) or 24 (right panel) hours and cell extracts were analyzed by immunoblot.

The sensitivity of HER2+ breast cancer cells to ERAD inhibition depends on continued protein synthesis [25]. The protein synthesis inhibitors cycloheximide (CHX) and puromycin function by interfering with the translocation step in protein synthesis and by inducing premature chain termination during translation, respectively. DDA activation of the ER stress response was reduced if protein synthesis was inhibited using either CHX or Puromycin over 8 hours (Figure 2A). Inhibition of protein synthesis with CHX also partly overcame PARP cleavage and Akt dephosphorylation, suggesting that induction of ER stress may be partially responsible for these DDA responses. In contrast, CHX did not overcome DDA-mediated downregulation of EGFR or HER3. Similar results were obtained after a 24 hour treatment period (Figure 2B), although CHX blockade of RBF3-induced PARP cleavage was less apparent, and CHX partially restored HER2 expression in BT474 cells. Results at shorter time points (2-16 hrs) showed that CHX rapidly and persistently blocked RBF3-mediated UPR (Figure 2C). A range of CHX concentrations were tested for their ability to reverse DDA responses. The results indicated that a complicated relationship exists between RBF3 responses and inhibition of protein synthesis (Figure 2D). A likely explanation for this result is that on the one hand UPR stress is associated with inhibition of protein synthesis through the PERK-eIF2α branch, and would suppress ER stress induced by misfolding of proteins such as HER1-3, while on the other hand, resolution of ER stress requires the synthesis of proteins including ATF4, CHOP, and GRP78. Overall, the results in Figure 2A–2D indicate that blockade of protein synthesis with CHX suppresses several DDA responses in a time- and concentration-dependent manner.

### EGFR or HER2 overexpression sensitizes cancer cells to DDA actions

HER3 plays a major role in the survival of HER2+ breast cancers and their resistance to HER2-targeted drugs [20, 21]. It was previously shown that breast cancer cells engineered to overexpress EGFR are sensitized to DDA-induced cell death and Akt dephosphorylation [33], but differential sensitivity to DDA-mediated HER3 downregulation was not examined in that report. T47D cells engineered to overexpress EGFR or HER2 and treated with EGF, the DDA NSC624205, or EGF + NSC624205 showed that EGFR or HER2 overexpression decreased basal HER3 expression (Figure 2E). This decreased baseline, combined with DDA treatment, reduced HER3 expression to very low levels. EGFR overexpression potentiated ER stress as measured by XBP1s and GRP78 expression at an intermediate (18 hr) time point (Figure 2F). Analysis after 15 hours showed that RBF3 had largely downregulated HER2 and HER3 in the EGFR overexpressing cells at this time point, while the levels of these proteins was unchanged in the vector control cells (Figure 2G). The ATF4 and XBP1s UPR markers were higher in the EGFR expressing line compared to the control, while RBF3 induced GRP78 to similar levels in both lines at 15 hours post treatment. Akt dephosphorylation was slightly enhanced in the context of EGFR overexpression at this time point. The differential effects of RBF3 on EGFR-overexpressing versus control cells on HER2, HER3, and phospho-Akt was amplified at 24 hours as compared with 15 and 18 hours. In contrast, at 24 hours after RBF3 treatment XBP1s levels were higher in the control cells than the EGFR overexpressing line. The ER stress response frequently peaks and then becomes weaker over time after ER chaperones have been upregulated, protein synthesis has been suppressed, and protein misfolding becomes resolved. Thus, the differences between time points likely results from the peak of the ER stress response occurring earlier in the EGFR overexpressing cells as compared with the control cells. HER4 was not expressed at detectable levels in the MDA-MB-468 or BT474 cell lines, but HER4 was expressed in the T47D line. RBF3 induced HER4 downregulation at the 24 hour time point, but not at the 15 hour time point.

### DDA effects on HER1-3 and Akt are separable from effects on the ER stress response

**Figure 3:**
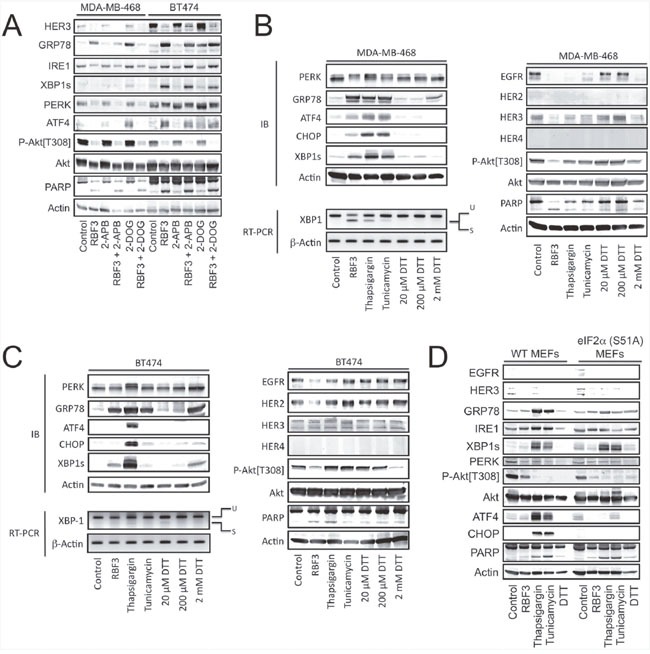
DDA activation of UPR is separable from effects on HER1-3 levels and Akt phosphorylation **(A)** MDA-MB-468 and BT474 cells were treated for 24 hours with 20 μM RBF3, 100 μM 2-Aminoethoxydiphenyl Borate (2-APB), or 4 mM 2-deoxyglucose (2-DOG) in the indicated combinations and cell extracts were analyzed by immunoblot. **(B)** MDA-MB-468 cells were treated for 24 hours with 20 μM RBF3, 400 nM thapsigargin, 500 ng/ml tunicamycin or dithiothreitol (DTT) at the indicated concentrations and cell extracts were analyzed by immunoblot. The effects of these same treatments on the splicing of the mRNA coding for XBP1s was assessed by reverse transcription of mRNA followed by DNA amplification (RT-PCR). **(C)** BT474 cells were treated for 24 hours with 20 μM RBF3, 400 nM thapsigargin, 500 ng/ml tunicamycin, or DTT at the indicated concentrations, and cell extracts were analyzed by immunoblot. The effects of these same treatments on the splicing of the mRNA coding for XBP1s was assessed by reverse transcription of mRNA followed by DNA amplification (RT-PCR). **(D)** Wild type or eIF2α[S51A] double knock-in mutant MEFs were treated for 24 hours with 20 μM RBF3, 400 nM thapsigargin, 500 ng/ml tunicamycin, 5 mM DTT, or vehicle and cell extracts were prepared and analyzed by immunoblot.

The DDA RBF3 was compared with 2-deoxyglucose (2-DOG), thapsigargin, tunicamycin, and dithiothreitol (DTT) to determine whether downregulation of HER-family receptors, decreased Akt phosphorylation, and induction of cell death is common among all ER stress inducers. 2-DOG strongly activated UPR in both the MDA-MB-468 and BT474 lines, but did not cause downregulation of either HER3 expression or Akt phosphorylation, and did not increase cell death as measured by PARP cleavage (Figure 3A). Some ER stress responses result from increased cytoplasmic Ca^2+^ mediated through IP_3_ receptors. To evaluate the role of this mechanism in RBF3 actions, we employed the IP_3_R antagonist 2-aminoethoxydiphenyl borate (2-APB). 2-APB reduced RBF3-mediated GRP78 upregulation, but did not alter RBF3-induced downregulation of HER3, Akt dephosphorylation, or PARP cleavage. Comparison of RBF3 with thapsigargin, tunicamycin, or DTT treatment of MDA-MB-468 cells revealed that RBF3 most effectively upregulated GRP78 expression and IRE1-dependent XBP1 mRNA processing, while thapsigargin and tunicamycin elevated ATF4 and CHOP expression more effectively than RBF3 (Figure 3B). In this cell line, 2 mM DTT only weakly activated the ER stress response as measured by GRP78 upregulation. EGFR was downregulated by all of the ER stressors. HER3 levels were particularly sensitive to 20 μM RBF3 and less so to 2 mM DTT. Of all of the ER stress inducers, only RBF3 induced significant PARP cleavage, and RBF3 most strongly downregulated Akt phosphorylation.

Although BT474 cells responded somewhat differently to the ER stressors than the MDA-MB-468 cells, RBF3 and DTT increased XBP1s and GRP78 expression with little upregulation of ATF4 or CHOP expression (Figure 3C). This is in contrast to thapsigargin treatment, which strongly upregulated both ATF4 and CHOP, and more strongly decreased PERK electrophoretic mobility, consistent with its increased phosphorylation [37]. Under these conditions, only RBF3 strongly downregulated EGFR and HER2 expression, while both RBF3 and DTT, but none of the other ER stressors, decreased Akt phosphorylation. Taken together, the results in Figure 3A–3C demonstrate that RBF3 produces a pattern of ER stress response that is different from that observed with 2-DOG, thapsigargin, and tunicamycin. RBF3 responses were most similar to those seen with DTT, although DTT was applied to the cells at a 100 times higher concentration than RBF3. RBF3 and DTT decrease Akt phosphorylation in both cell lines. RBF3 reduces HER-family receptor expression in both cell lines, while DTT only does so in the MDA-MB-468 line. Thus, the DDA RBF3 is unique when compared to ER stressors 2-DOG, thapsigargin, tunicamycin, and DTT with respect to the spectrum of ER stress responses, Akt dephosphorylation, HER-family receptor downregulation, and cell death induction.

In contrast to the robust effects of RBF3 on the EGFR+ or HER2+ breast cancer cell lines, RBF3 and DTT did not induce an ER stress response in wild type Mouse Embryo Fibroblasts (MEFs) and weakly suppressed Akt phosphorylation and induced PARP cleavage (Figure 3D). However, thapsigargin and tunicamycin induced a robust UPR, markedly suppressed Akt phosphorylation, and strongly upregulated PARP cleavage. In MEFs in which eIF2α with the PERK phosphorylation site Ser51 mutated to Ala was heterozygously knocked in [38], CHOP, ATF4, and GRP78 upregulation by thapsigargin and tunicamycin was significantly blunted. However, the S51A eIF2α mutation did not affect thapsigargin- or tunicamycin-induced PARP cleavage or XBP1s upregulation. MEFs express very low levels of the HER-family receptors and this may contribute to their relative resistance to thiol-reactive agents such as RBF3 and DTT.

### Cooperation between DDAs and receptor tyrosine kinase inhibitors

**Figure 4:**
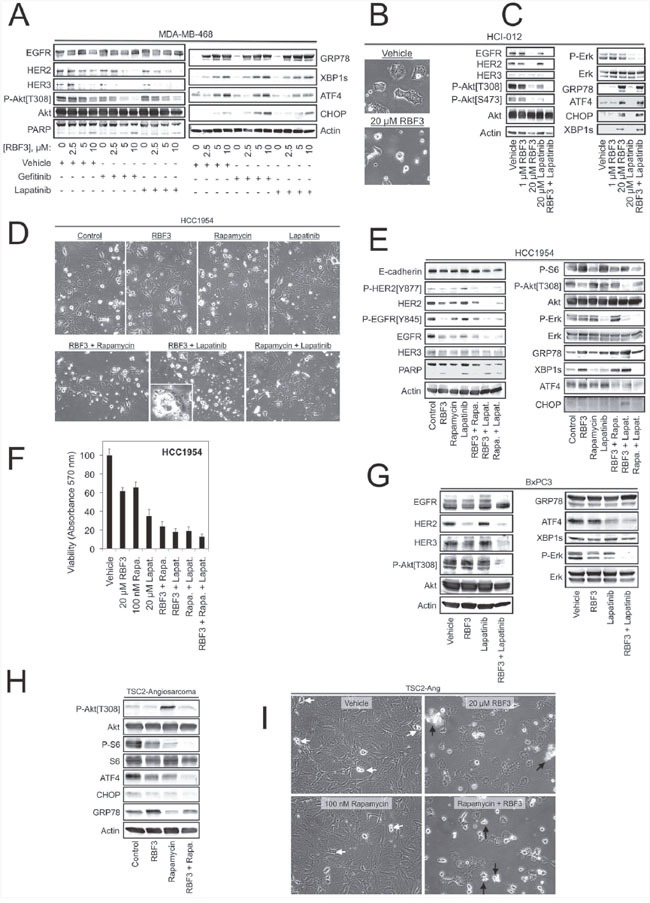
DDAs may be useful in combination therapies for combating resistance to mTORC1-, EGFR-, or HER2-targeted agents **(A)** MDA-MB-468 cells were treated for 24 hours with RBF3 alone at the indicated concentrations, or RBF3 combined with 2.5 μM Gefitinib or 2.5 μM Lapatinib, and cell extracts were prepared and analyzed by immunoblot. **(B)** Micrographs of the HCI-012 cell line after a 24 hour treatment with vehicle or 20 μM RBF3. **(C)** Immunoblot analysis of HCI-012 cell extracts after the indicated 24 hour treatments. **(D)** Micrographs of HCC1954 cells after 24 hour treatments with 20 μM RBF3, 100 nM rapamycin, 20 μM Lapatinib, or the indicated drug combinations. **(E)** Immunoblot analysis of HCC1954 cells treated as in Figure 4D. **(F)** Viability of HCC1954 cells after 24 hours of the indicated treatments using the MTT assay. Results are presented as the mean ± standard deviation of triplicate determinations. **(G)** Immunoblot analysis of BxPC3 pancreatic cancer cell extracts after a 24 hour treatment with 20 μM RBF3, 20 μM Lapatinib, or the two drugs combined. **(H)** Extracts from TSC2-Ang1 cells treated with 20 μM RBF3, 100 nM rapamycin, or RBF3 + rapamycin were analyzed by immunoblot. **(I)** Micrographs of TSC2-Ang1 cells treated as in Figure 4H. White arrows denote cells undergoing division. Black arrows indicate cell death.

Since DDAs and receptor tyrosine kinase (RTK) inhibitors such as EGFR-specific Gefitinib and EGFR/HER2-specific Lapatinib block the functions of EGFR and HER2 through distinct mechanisms, we examined whether these two classes of agents cooperate to inactivate mitogenic signaling pathways and activate UPR. Co-treatment with either 2.5 μM Gefitinib or Lapatinib lowered the concentration of RBF3 required to downregulate HER3 levels and Akt phosphorylation (Figure 4A). Under these conditions, the combination treatments did not alter UPR as measured by GRP78, XBP1s, or ATF4 expression, but Gefitinib, and to a lesser extent Lapatinib, cooperated with RBF3 to upregulate CHOP expression.

The MDA-MB-468 and BT474 cell lines are well characterized models for EGFR and HER2 overexpressing breast cancer, but form highly homogenous tumors with questionable relevance to human breast cancer. Patient-Derived Xenograft (PDX) models are the system of choice for studying the effectiveness of anticancer agents against human breast cancers (reviewed in [39]), but the cellular heterogeneity responsible for their higher clinical relevance renders studies of the mechanisms of drug action on cancer cells difficult. To bridge this gap, we isolated a cell line from the previously described HCI-012 HER2+ and ER-, PR- PDX line [40] using Conditional Cell Reprogramming (CCR) [41, 42]. The HCI-012 cell line formed tumors when injected into the mammary fat pads of immunocompromised NOD-SCID-γ (NSG) mice at 100% efficiency (n = 5/5), and the heterogeneous morphology of the resulting tumors was similar to that of the parental xenograft line (Supplementary Figure 3A). The HCI-012 cells rapidly initiate cell death if not cultured in the CCR medium (Supplementary Figure 3B), consistent with previous reports that the CCR approach maintains reversible immortality of epithelia-derived cell lines *in vitro* [42]. RBF3 treatment of HCI-012 cells induced cell death (Figure 4B), which was associated with upregulation of ER stress markers, reduced Akt phosphorylation, but RBF3 had no effect on Erk phosphorylation (Figure 4C). Lapatinib partially reduced Akt phosphorylation, and strongly suppressed ERK phosphorylation, but did not alter EGFR, HER2, or HER3 levels, nor did it alter the expression of ER stress markers. The combination of RBF3 and Lapatinib suppressed EGFR and HER2 expression and completely abrogated both Akt and Erk phosphorylation. This result suggests that these two agents are complementary in their effects on mitogenic/survival signaling. In the HCI-012 cells, Lapatinib did not influence RBF3 upregulation of the ER stress markers GRP78, ATF4, XBP1s, or CHOP.

### DDA impacts pathways that mediate resistance to HER2- and mTORC1-targeted therapeutics

The HCC1954 cell line is a model of Trastuzumab resistant, HER2-positive breast cancer, and resistance is thought to be mediated by the activating Phosphatidylinositol 3-kinase (PI3K) mutation H1047R [43]. Observation of cultures revealed that combining RBF3 and Lapatinib resulted in the highest level of cell death (Figure 4D). Under these conditions, RBF3 and Lapatinib cooperated to downregulate EGFR and HER2, to increase fractional PARP cleavage, and to suppress Akt phosphorylation (Figure 4E). The mTORC1 inhibitor rapamycin did not cooperate with RBF3 to produce these effects and antagonized RBF3-mediated Akt dephosphorylation. Lapatinib only weakly potentiated RBF3-induced UPR with respect to GRP78, XBP1s, or ATF4 levels, but cooperated with RBF3 to upregulate CHOP expression. RBF3 + Lapatinib was more effective in reducing HCC1954 cell viability than either of the compounds applied individually (Figure 4F).

Previous studies demonstrated that in contrast to EGFR or HER2 overexpressing breast cancer lines, the BxPC3 pancreatic cancer cell line is refractory to DDAs [33]. Challenging BxPC3 cells with RBF3 indicated that it reduced HER2 expression, but had little effect on the levels or phosphorylation states of the other proteins examined (Figure 4G). Lapatinib had no significant effect on HER1-3 expression, or Akt or Erk phosphorylation. However, RBF3 + Lapatinib not only downregulated HER2, but also strongly downregulated HER3, and suppressed both Akt and Erk phosphorylation.

mTORC1 inhibitors such as the rapamycin analogs (rapalogs) inadvertently activate the PI3K/Akt axis by removing negative feedback mediated through S6K1 [44, 45]. Since Akt activation might detract from the clinical utility of rapalogs, which are used in immunosuppression, the treatment of human cancers, and the management of Tuberous Sclerosis (TSC) (Reviewed in [46]), the reversal of rapamycin-mediated Akt activation by RBF3 was examined. In TSC, individuals have mutations in the genes coding for the proteins TSC1 or TSC2 and develop benign tumors in multiple tissues in part because the TSC1/TSC2 complex is a GTPase activating protein for the Rheb GTPase responsible for mTORC1 activation (reviewed in [47]). Thus, mTORC1 activation is characteristic of TSC. Rapalogs are FDA-approved for TSC treatment, but activation of Akt could be a significant side effect. To address this point, angiosarcoma cells from a TSC2 knockout mouse (TSC2-Ang1; ATCC CRL-2620) were used as a model system. Treatment of these cells with RBF3 had little effect on ER stress markers, which were high under control conditions (Figure 4H). Rapamycin strongly increased Akt phosphorylation and co-administration of RBF3 reduced Akt phosphorylation to basal levels. TSC2-Ang1 cell death was only observed upon treatment with RBF3 or RBF3 + Rapamycin (black arrows), whereas vehicle and rapamycin treated cells continued to proliferate (white arrows) (Figure 4I). The combination of RBF3 and rapamycin more effectively suppressed S6 phosphorylation than rapamycin alone. The results in Figure 4 suggest that DDA combinations with RTK inhibitors might provide improved anticancer actions. Pairing DDAs with rapalogs may both increase mTORC1 inhibition and prevent off-target Akt activation.

### Preparation and characterization of multivalent DDAs

**Figure 5:**
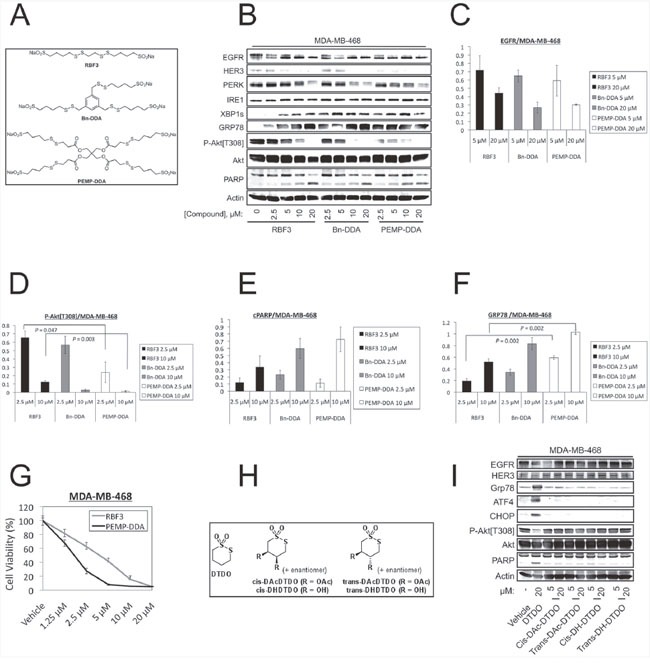
Increasing the number of pharmacophores per DDA molecule improves potency against MDA-MB-468 cells **(A)** Structure of bivalent DDA RBF3 and novel trivalent (Bn-DDA) and tetravalent (PEMP-DDA) DDAs. **(B)** Immunoblot analyses of MDA-MB-468 cells treated with the indicated concentrations of DDAs for 24 hours. The results in panel B were replicated a total of three times, and quantified with respect to changes in EGFR levels, Akt phosphorylation on Thr^308^, PARP cleavage (cPARP), and GRP78 expression in panels (C, D, E, and F) respectively. Statistically significant differences are denoted with *P*-values. **(G)** MTT assays performed on cells treated with the indicated concentrations of DDAs for 72 hours. **(H)** Structural alterations to the parent cyclic, monovalent DDA, DTDO. **(I)** Extracts from MDA-MB-468 cells treated with the DTDO or its derivatives for 24 hours at the specified concentrations were analyzed by immunoblot.

DDA RBF3 contains two repeats of the previously defined pharmacophore [33]. New DDAs, termed Bn-DDA and PEMP-DDA, containing three and four copies of the pharmacophore per molecule, respectively, were synthesized to determine whether they have increased potency over RBF3 (Figure 5A). Treatment of the DDA sensitive EGFR+ MDA-MB-468 cell line with increasing concentration of each compound indicated that PEMP-DDA decreased Akt phosphorylation and HER3 levels more than RBF3 or Bn-DDA (Figure 5B). This immunoblot analysis was repeated a total of three times and DDA-induced changes in EGFR, phospho-Akt[Thr308], PARP cleavage (cPARP), and GRP78 levels were plotted in Figure 5C, 5D, 5E, and 5F, respectively. The replicate immunoblot analyses are shown in Supplementary Figure 1A-1D. Statistically significant differences are indicated with *P*-values obtained using Student's unpaired *t*-test. All bands were normalized to the corresponding Actin loading control before the ratios between drug treatments were calculated. MTT assays with increasing concentrations of RBF3 and PEMP-DDA showed that both reduced cell viability in a concentration-dependent manner (Figure 5G).

DTDO is a cyclic form of the previously identified DDA pharmacophore [33]. Since the two sulfur atoms of DTDO are involved in DDA chemistry, we examined whether derivatization of the second and third carbon atoms of the four-carbon linker by either hydroxyl or acetyl groups (Figure 5H) altered DDA actions on cells. DTDO (20 μM) reduced activating Akt phosphorylation, upregulated markers of ER stress, and increased PARP cleavage (Figure 5I). In contrast, the hydroxylated or acetylated DTDO derivatives with either *cis* or *trans* configurations had little or no effect on these endpoints at 20 μM. This result suggests that the cyclic DDAs act through similar mechanisms as the linear forms (e.g., RBF3).

**Figure 6:**
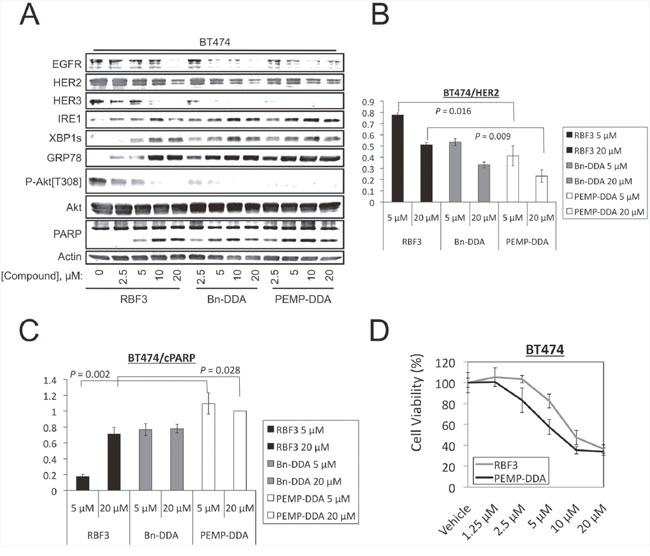
Increasing the number of pharmacophores per DDA molecule improves potency against BT474 cells **(A)** Immunoblot analyses of BT474 cells treated with the indicated concentrations of DDAs for 24 hours. The results in panel A were replicated a total of three times, and quantified with respect to changes in HER2 levels and PARP cleavage (cPARP) in panels (B and C) respectively. Statistically significant differences are denoted with *P*-values. **(D)** MTT assays performed on cells treated with the indicated concentrations of DDAs for 72 hours.

Experiments similar to those carried out with MDA-MB-468 cells in Figure 5 were carried out with the HER2+, DDA-sensitive BT474 cell line in Figure 6. All three DDAs decreased EGFR, HER2, and HER3 expression, increased PARP cleavage, reduced Akt phosphorylation and upregulated the ER stress markers GRP78 and XBP1s (Figure 6A). PEMP reduced HER2 expression (Figure 6B) and PARP cleavage (Figure 6C) significantly more than RBF3 at the same drug concentrations. PEMP-DDA also reduced BT474 cell viability more than RBF3 (Figure 6D).

The DDA responsive, HER2+ SKBR3 cell line produced similar responses to bi-, tri-, and tetra-functional DDAs as observed with the MDA-MB-468 and BT474 lines (Supplementary Figure 2A). As expected, the DDA-resistant MDA-MB-231 and HCC1954 exhibited ER stress in control samples and did not exhibit a response to any of the DDAs (Supplementary Figure 2B and 2C, respectively).

### DDAs are not toxic to cardiomyocytes or MCF10/DCIS cells

**Figure 7:**
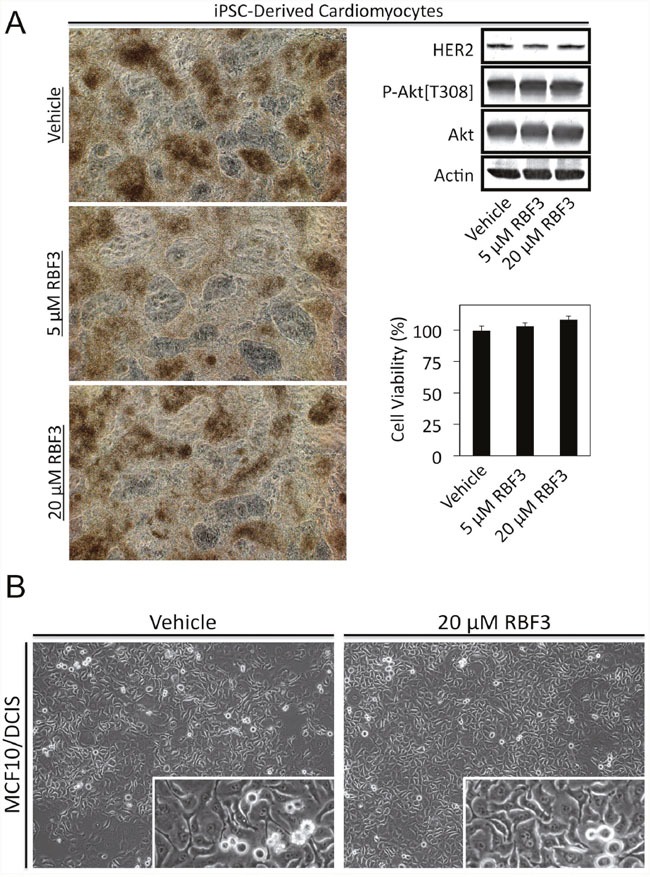
DDAs are not toxic to cardiomyocytes or MCF10/DCIS cells **(A)** Cardiomyocytes differentiated from iPSCs were treated as indicated for 24 hours and photographed (left panel). Videos demonstrating beating of the cardiomyocytes are presented in Supplementary Videos Supplementary Video 1–Supplementary Video 3. Cardiomyocytes treated in parallel were subjected to immunoblot analysis with the indicated antibodies (top right panel), and MTT cell viability assays (bottom right panel). **(B)** Photomicrographs of MCF10/DCIS cells treated for 24 hours as indicated. Higher magnification insets show ongoing cell division.

Cardiotoxicity is a side effect of the HER2 specific monoclonal antibody Trastuzumab. Therefore we examined whether RBF3 altered the behavior of cardiomyocytes differentiated from human induced Pluripotent Stem Cells (iPSCs) as described previously [48, 49]. Microscopic examination of cardiomyocytes treated for 24 hours with RBF3 did not change their appearance (Figure 7A, left panel) and their rate of beating was not altered (Supplementary Videos, Supplementary Video 1–Supplementary Video 3). Immunoblot analysis demonstrated that the cardiomyocytes expressed HER2, but RBF3 treatment did not decrease the levels or HER2 or suppress Akt phosphorylation (Figure 7A, upper right panel). MTT assays showed that RBF3 did not reduce the viability of cardiomyocytes (Figure 7A, lower right panel).

The MCF10/DCIS cell line serves as a model of Ductal Carcinoma *in situ* in which cancer cells aberrantly proliferate, but are unable to invade through the basement membrane to invade locally. MCF10/DCIS cells are considered to express normal levels of EGFR and HER2 [50]. MCF10/DCIS cells treated for 24 hours with 20 μM RBF3 did not die but continued to proliferate (Figure 7B).

## DISCUSSION

Previous work showed that cancer cell death caused by DDAs correlates with HER1-3 downregulation and Akt dephosphorylation [33]. The results presented here extend these findings by showing that DDAs also activate UPR. We previously demonstrated the ability of DDAs to break disulfide bonds in the model compound oxidized Glutathione (GSSG) [33]. Disulfide bond formation is a critical component of the folding of both integral membrane and secreted proteins, and interference with this process by treatment with reducing agents such as dithiothreitol (DTT) activates UPR [51, 52]. The results in Figure 1 demonstrate that DDAs activate all three branches of the ER stress response. Interestingly, DDA RBF3 activates UPR at low micromolar concentrations, while millimolar concentrations of DDT are required to induce a similar level of ER stress. It is tempting to speculate that this striking difference in potency relates to the structural uniqueness of DDAs in having a nucleophilic sulfinate group, an electrophilic disulfide group, and the ability of the pharmacophore to interconvert between cyclic and linear forms. Alternately, the bifunctional nature of DDAs may render them more difficult for cells to neutralize than DTT or similar reducing agents.

The observation that DDAs act through mechanisms involving UPR, Akt inactivation, and HER1-3 downregulation raises the question of which of these pathways contributes to DDA anticancer actions, and whether these responses are mechanistically related. A comparison of RBF3 with other ER stress inducers and the use of CHX to block protein synthesis and ER stress provide some insight into these issues. Thapsigargin upregulates ATF4, XBP1s, and CHOP expression more strongly than RBF3 in MDA-MB-468 cells, while RBF3 more effectively suppresses Akt phosphorylation than either tunicamycin or thapsigargin (Figure 1D). Like 20 μM RBF3, 2 mM DTT induces downregulation of EGFR and HER3 and suppresses Akt phosphorylation, but under these conditions only weakly induces PARP cleavage and GRP78 expression, and does not upregulate ATF4, CHOP, or XBP1s protein expression or XBP1 mRNA splicing (Figure 3B). Interestingly, 2-DOG strongly induces ER stress in MDA-MB-468 and BT474 cells as measured by upregulation of GRP78 and ATF4, but does not induce PARP cleavage, or suppress Akt phosphorylation (Figure 3A). Further, 2-DOG does not suppress HER3 expression in either the MDA-MB-468 or BT474 cell lines. This suggests that reduction of HER1-3 receptor expression, suppression of Akt phosphorylation, and increased PARP cleavage relate to the thiol reactivity of RBF3 and DTT rather than to induction of UPR alone. However, results obtained with protein synthesis inhibitors show that while blockade of translation overcomes the ability of RBF3 to activate UPR and partially overcome PARP cleavage, this treatment did not prevent RBF3-mediated downregulation of HER1-3 expression (Figure 2A–2D). Overall, the results of these experiments obtained with the use of 2-DOG and DTT suggest that the ability of RBF3 to induce cancer cell death results from a combination of UPR activation, HER1-3 downregulation, and decreased Akt phosphorylation.

Ideally, cancer therapeutic agents should be toxic to cancer cells with little or no impact on normal cells. The principles of “oncogene addiction [1, 53]” and “synthetic lethality [54, 55]” are strategies to realize this ideal. These approaches are exemplified by the use of BCR-Abl inhibitors for the treatment of Chronic Myelogenous Leukemia (CML), HER2-directed monoclonal antibodies and tyrosine kinase inhibitors for the treatment of HER2+ breast tumors, and PARP inhibitors for the treatment of BRCA1/2-mutant ovarian cancers. However, these approaches suffer from cancer “escape” from therapy through a variety of mechanisms. Thus, in many cases cancer cures may require multiple drugs to overcome both the driver oncogene and potential resistance mechanisms, or the discovery of multifunctional anticancer drugs that target the appropriate mechanisms.

Breast cancers devoid of Estrogen Receptor (ER-), Progesterone Receptor (PR-), and HER2 expression (HER2-) are termed Triple-Negative Breast Cancers. Currently, no targeted therapies for TNBCs exist. EGFR has been suggested as a therapeutic target for TNBCs [56, 57] and it has been estimated that up to 50% of TNBCs may overexpress EGFR at the protein level [28]. The potential for the use of DDAs against TNBCs is supported by the observation that the EGFR overexpressing MDA-MB-468 TNBC cell line is the most sensitive line to DDAs identified to date.

DDAs are selectively cytotoxic to breast cancer cells that overexpress either HER2 or EGFR and EGFR overexpression potentiates DDA-induced Akt dephosphorylation [33]. In the present study we examined whether EGFR overexpression also potentiates other DDA responses including HER3 downregulation and activation of ER stress. HER3 mediates a number of resistance mechanisms to HER2-targeted therapies through its ability to be phosphorylated by EGFR, IGF-1R, and c-MET [21, 58–61] and activate the PI3K/Akt pathway. In the T47D ER+ breast cancer cell line ectopic expression of either EGFR or HER2 rendered endogenous HER3 more sensitive to downregulation by the DDA NSC624205 (Figure 2E), and EGFR overexpression sensitizes EGFR, HER2, and HER3 to RBF3-mediated downregulation (Figure 2G).

A concern with DDAs relates to their ability to break disulfide bonds and potentially alter the function of multiple secreted or membrane proteins. A number of cell types that express normal levels of EGFR and HER2, such as T47D, MCF10/DCIS, MEF lines are unaffected by DDAs. However, T47D cells become responsive to the toxic effects of DDAs upon overexpression of EGFR or HER2 ([33] and herein (Figure 2E–2G).

Since a side effect of Trastuzumab is cardiotoxicity, the possibility that DDAs might also be cardiotoxic is a concern. The results presented in Figure 7 indicate that while the cardiomyocytes expressed high levels of HER2, there was no effect of RBF3 on HER2 levels in contrast to what is observed in cancer cells. Further, RBF3 had no effect on the beating of the cardiomyocytes in culture (see videos Supplementary Video 1-Supplementary Video 3 in supplemental material). We speculate that the reason that DDAs do not downregulate HER2 in cardiomyocytes is that in these cells HER2 is expressed at normal levels rather than being overexpressed. Therefore the addition of DDAs does not cause sufficient ER stress to kill cardiomyocytes. This is consistent with the observation that nearly all breast cancers express HER2, but DDAs are only toxic to the lines that exhibit dramatic HER2 or EGFR overexpression.

**Figure 8:**
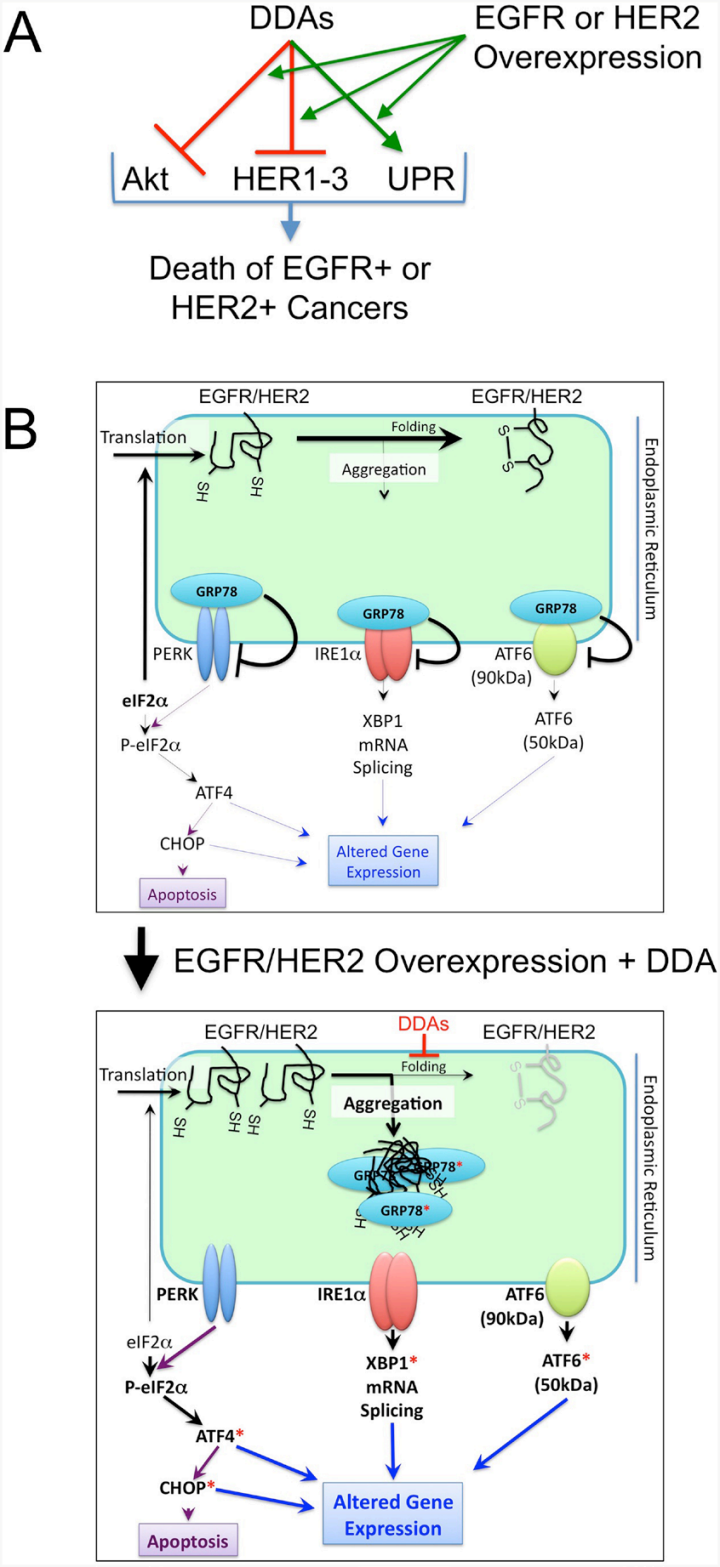
Model for the anticancer actions of DDAs **(A)** DDAs function to suppress tumor cell division and survival through mechanisms involving suppression of Akt phosphorylation, downregulation of EGFR (HER1), HER2, and HER3, and activation of UPR. Overexpression of EGFR or HER2 potentiates each of these mechanisms. **(B)** At normal expression levels, EGFR and HER2 are folded efficiently and do not induce ER stress (upper panel). Overexpression of EGFR or HER2 cooperates with DDA treatment, which blocks disulfide bond formation and protein folding, to activate UPR (lower panel). Red asterisks denote ER stress markers examined experimentally in Figures 1-6.

Because of the large number of disulfide bonds in the HER-family receptor cysteine-rich extracellular domains and the ability of DDAs to break disulfide bonds, we hypothesize that overexpression of HER-family receptors such as EGFR selectively exacerbate the ER stress induced by DDAs. Consistent with this expectation, EGFR overexpression in T47D cells potentiated RBF3-induced ER stress and this effect was particularly notable at early time points. We propose the model for DDA action in Figure 8A where DDAs selectively induce the death of EGFR+ and HER2+ cancers through the suppression of Akt phosphorylation, downregulation of HER1-3 expression, and activation of UPR. DDA induction of UPR and Akt dephosphorylation are potentiated by overexpression of EGFR, or to a lesser extent, HER2. Due to their unique and multifunctional mechanisms of action, DDAs may be well suited for targeting the pathways responsible for resistance to HER2- and EGFR-targeted agents and prove to be complementary to other therapeutic modalities including monoclonal antibodies and receptor tyrosine kinase inhibitors targeting HER-family oncogenes. The high sensitivity of EGFR or HER2 overexpressing cancer cells to DDAs may derive from the large number of disulfide bonds in these proteins combined with the ability of DDAs to prevent the formation of Disulfide bonds in the ER (Figure 8B). In addition to examining DDA effects on breast tumors with overexpression of wild type EGFR or HER2, in future studies it will be important to determine whether mutants or splice variants of these proteins, such as HER2-delta 16 [62–64] are responsive to DDAs.

Cyclic and linear forms of the DDA pharmacophore can interconvert, and elicit similar cellular responses. Slight structural modifications made to either form of the DDA pharmacophore result in loss of biological activity ([33] and Figure 5H, 5I), but increasing the number of pharmacophores per molecule elevates DDA potency (Figure 5A-5G and Figure 6A–6D). This suggests a modular lead optimization approach in which improvements are made to the activity of the pharmacophore structure, and the optimized pharmacophore is then appended to a polyvalent scaffold to further increase DDA potency. These efforts are currently ongoing in our laboratories.

## MATERIALS AND METHODS

### Cell lines, construction of stable cell lines, and recombinant adenoviruses

The following cell lines were purchased from American Type Culture Collection (ATCC) (Manassas, VA): MDA-MB-468, BT474, T47D, SKBR3, MDA-MB-231, HCC1954, HEK 293, TSC2-Ang1, and BxPC3. The HCI-012 cell line was derived from the HCI-012 Patient-Derived Xenograft tumor line provided by Dr. Alana Welm [40] using conditional cell reprogramming [41, 42, 65]. Characterization of the HCI-012 cell line is shown in Supplementary Figure 3. Wild type and eIF2α[S51A] homozygous knock-in Mouse Embryo Fibroblasts (MEFs) were described previously [38].

Recombinant retroviruses were prepared and used to produce stable cell lines as described previously [66, 67]. Retroviral vectors encoding EGFR (plasmid 11011, [68]) and HER2 (plasmid 40978 [69]) were from Addgene (Cambridge, MA).

### Cell culture, preparation of cell extracts, and immunoblot analysis

Cells were grown in Dulbecco's modified Eagle's medium (GE Healthcare Life Sciences Logan, UT) supplemented with 10% fetal bovine serum (10% FBS-DMEM) in a humidified 37°C incubator with 5% CO_2_. Cell lysates were prepared using a buffer containing 1% Triton X100, 20 mM HEPES (pH 7.4), 1 mM EDTA, 1 mM EGTA, 0.1% β-mercaptoethanol, 5% glycerol, 10 nM microcystin, 200 μM Na_3_VO_4_ and 40 mM Na_2_H_2_P_2_O_7_ as described previously [70].

Immunoblot analysis was carried out using primary antibodies purchased from Santa Cruz Biotechnology (Dallas, TX) [Actin, sc-1616-R; ERK, sc-93; JNK, sc-572; P-JNK, sc-6254; Src, sc-18; EGFR, sc-03; GRP78, sc-13539; Phosphotyrosine (PY99), sc-7020], Cell Signaling Technology (Beverly, MA) [Akt, #4691; P-Akt[T308], #13038; P-Akt[S473], #9271; ATF4, #11815; CHOP, #2895; P-Src[Y527], #2105; EGFR, #4267; HER2, #2165; HER3, #4754; Calnexin, #2679; #13024;P-Erk, #9101; IRE1α, #3294; XBP1s, #12782; PARP, #9532; PDI, #3501; PERK, #5683; S6, #2212; P-S6, #2211; P-Src[Y416], #6943], BD Transduction Laboratories (San Jose, CA) [PAI-1, 612024], Millipore (Temecula, CA) [anti-phosphotyrosine (4G10), 05-321], and Sigma-Aldrich (St. Louis, MO) [anti-FLAG (M2), F3165]. To quantify immunoblot results, bands were analyzed using ImageJ (https://imagej.nih.gov/ij/) and each band was normalized to the corresponding Actin loading control band.

EGF (GF001) was obtained from Chemicon International (Temecula, CA). Lapatinib (sc-202205) was from Santa Cruz Biotechnology. NSC624205 was a gift from the National Cancer Institute's Developmental Therapeutics Program. The following reagents were purchased from the indicated sources: tunicamycin, 2-deoxyglucose: Sigma-Aldrich (St. Louis, MO); 2-aminoethoxydiphenyl borate (2-APB): StressMarq Biosciences (Cadboro Bay, Victoria, Canada); thapsigargin: AdipoGen (San Diego, CA); Puromycin, Rapamycin, Cycloheximide: EMD Biosciences (Darmstadt, Germany); Gefitinib, Lapatinib, SAHA: Selleck Chemicals (Houston, TX); dithiothreitol: Fisher Scientific (Pittsburgh, PA).

### Transfection of HEK293 cells and luciferase assays

Transfections were performed using Lipofectamine Reagent (Invitrogen, Carlsbad, CA) according to the manufacturer's instructions. Cells were incubated for 48 hours after transfection, and cell extracts analyzed by luciferase assays, with background readings subtracted from the luciferase assay values. Relative Luminescence Units (RLUs) were normalized to the number of micrograms of protein assayed. The results are presented as the mean of triplicate determinations ± standard deviation. The ATF6 Reporter (plasmid 11976 [71]) and FLAG-ATF6α (plasmid 11975 [72]) constructs were obtained from Addgene.

### MTT cell viability assays

Cell viability was evaluated using MTT (3-(4,5-dimethylthiazol-2-yl)-2,5-diphenyltetrazolium bromide) assays carried out based on the manufacturer's instructions (kit CGD1, Sigma-Aldrich, St. Louis, MO).

### Thymidine incorporation assays

Tritiated thymidine incorporation assays were performed as described previously [73]. Results are presented as the mean ± standard deviation of triplicate or quadruplicate determinations.

### RT-PCR

RNA was isolated from MDA-MB-468 and BT474 cells with Trizol Reagent (Invitrogen) according to the manufacturer's instructions, and reverse transcribed to cDNA under the following conditions: 25°C for 10 min, 42°C for 30 min, and 95°C for 5 min. PCR was performed using XBP1 and β-Actin primers. The primers used to amplify XBP1 are as follows: Forward: CCTGGTTGCTGAAGAGGAGG and Reverse: CCATGGGGAGATGTTCTGGAG. The primers used to amplify β-Actin are as follows: Forward: GGATGCAGAAGGAGATCAC and Reverse: AAGGTGGACAGCGAGGCCAG. Reactions were performed as follows: 96°C for 5 min followed by 35 cycles of 95°C for 45 sec, 60°C for 1 min, and 72°C for 30 sec. Reaction products were visualized on 3% agarose gels.

### Synthesis of DDAs

DDA synthesis and characterization is presented in Supplemental Material (Supplementary Information and Supplementary Figure 4).

### Cardiomyocyte differentiation

To induce cardiomyocyte differentiation we utilized the PSC Cardiomyocyte Differentiation Kit (ThermoFisher, Grand Island, NY). Briefly, human iPSCs were grown in feeder-free conditions using hES qualified Matrigel (Corning, Auburn, MI) and mTeSR1 medium (Stem Cell Technologies, Vancouver, BC, Canada). iPSC colonies were dissociated from one 35 mm dish using Gentle Cell Dissociation Reagent (Stem Cell Technologies) for eight minutes at 37°C to make a single cell suspension. The cells were divided equally among the wells of a 12-well plate coated with Matrigel using mTeSR1 medium and ROCK inhibitor (10 uM final concentration for the first 24 hours). Medium was changed daily with mTeSR1 until the iPSCs formed a monolayer of approximately 80% confluency. To induce mesoderm differentiation, Cardiomyocyte Differentiation Medium A was added for 48 hours (Days 0-2). For cardiac mesoderm specification, Cardiomyocyte Differentiation Medium B was added for the next 48 hours (Days 2-4). For cardiomyocyte maturation, cells were maintained in Cardiomyocyte Maintenance Medium for the duration of culture (Day 4+), replacing medium every other day. Spontaneous cell contraction began on day 10.

## SUPPLEMENTARY MATERIALS FIGURES









## References

[1] Jonkers J, Berns A (2004). Oncogene addiction: sometimes a temporary slavery. Cancer Cell.

[2] Weinstein IB, Joe AK (2006). Mechanisms of disease: Oncogene addiction--a rationale for molecular targeting in cancer therapy. Nat Clin Pract Oncol.

[3] Pagliarini R, Shao W, Sellers WR (2015). Oncogene addiction: pathways of therapeutic response, resistance, and road maps toward a cure. EMBO Rep.

[4] Baselga J, Tripathy D, Mendelsohn J, Baughman S, Benz CC, Dantis L, Sklarin NT, Seidman AD, Hudis CA, Moore J, Rosen PP, Twaddell T, Henderson IC (1999). Phase II study of weekly intravenous trastuzumab (Herceptin) in patients with HER2/neu-overexpressing metastatic breast cancer. Semin Oncol.

[5] Molina MA, Codony-Servat J, Albanell J, Rojo F, Arribas J, Baselga J (2001). Trastuzumab (herceptin), a humanized anti-Her2 receptor monoclonal antibody, inhibits basal and activated Her2 ectodomain cleavage in breast cancer cells. Cancer Res.

[6] Pegram MD, O’Callaghan C (2001). Combining the anti-HER2 antibody trastuzumab with taxanes in breast cancer: results and trial considerations. Clin Breast Cancer.

[7] McKeage K, Perry CM (2002). Trastuzumab: a review of its use in the treatment of metastatic breast cancer overexpressing HER2. Drugs.

[8] Blumenthal GM, Scher NS, Cortazar P, Chattopadhyay S, Tang S, Song P, Liu Q, Ringgold K, Pilaro AM, Tilley A, King KE, Graham L, Rellahan BL (2013). First FDA approval of dual anti-HER2 regimen: pertuzumab in combination with trastuzumab and docetaxel for HER2-positive metastatic breast cancer. Clin Cancer Res.

[9] Harbeck N, Beckmann MW, Rody A, Schneeweiss A, Muller V, Fehm T, Marschner N, Gluz O, Schrader I, Heinrich G, Untch M, Jackisch C (2013). HER2 Dimerization Inhibitor Pertuzumab - Mode of Action and Clinical Data in Breast Cancer. Breast Care (Basel).

[10] O’Sullivan CC, Swain SM (2013). Pertuzumab: evolving therapeutic strategies in the management of HER2-overexpressing breast cancer. Expert Opin Biol Ther.

[11] Johnston SR, Leary A (2006). Lapatinib: a novel EGFR/HER2 tyrosine kinase inhibitor for cancer. Drugs Today (Barc).

[12] Montemurro F, Valabrega G, Aglietta M (2007). Lapatinib: a dual inhibitor of EGFR and HER2 tyrosine kinase activity. Expert Opin Biol Ther.

[13] Tuma RS (2007). Lapatinib moves forward in inflammatory and early HER2-positive breast cancer trials. J Natl Cancer Inst.

[14] Blackwell KL, Pegram MD, Tan-Chiu E, Schwartzberg LS, Arbushites MC, Maltzman JD, Forster JK, Rubin SD, Stein SH, Burstein HJ (2009). Single-agent lapatinib for HER2-overexpressing advanced or metastatic breast cancer that progressed on first- or second-line trastuzumab-containing regimens. Ann Oncol.

[15] Knuefermann C, Lu Y, Liu B, Jin W, Liang K, Wu L, Schmidt M, Mills GB, Mendelsohn J, Fan Z (2003). HER2/PI-3K/Akt activation leads to a multidrug resistance in human breast adenocarcinoma cells. Oncogene.

[16] Nahta R, Esteva FJ (2006). HER2 therapy: molecular mechanisms of trastuzumab resistance. Breast Cancer Res.

[17] Valabrega G, Montemurro F, Aglietta M (2007). Trastuzumab: mechanism of action, resistance and future perspectives in HER2-overexpressing breast cancer. Ann Oncol.

[18] Liu L, Greger J, Shi H, Liu Y, Greshock J, Annan R, Halsey W, Sathe GM, Martin AM, Gilmer TM (2009). Novel mechanism of lapatinib resistance in HER2-positive breast tumor cells: activation of AXL. Cancer Res.

[19] Hu S, Fu W, Xu W, Yang Y, Cruz M, Berezov SD, Jorissen D, Takeda H, Zhu W (2015). Four-in-one antibodies have superior cancer inhibitory activity against EGFR, HER2, HER3, and VEGF through disruption of HER/MET crosstalk. Cancer Res.

[20] Claus J, Patel G, Ng T, Parker PJ (2014). A role for the pseudokinase HER3 in the acquired resistance against EGFR- and HER2-directed targeted therapy. Biochem Soc Trans.

[21] Xia W, Petricoin EF, Zhao S, Liu L, Osada T, Cheng Q, Wulfkuhle JD, Gwin WR, Yang X, Gallagher RI, Bacus S, Lyerly HK, Spector NL (2013). An heregulin-EGFR-HER3 autocrine signaling axis can mediate acquired lapatinib resistance in HER2+ breast cancer models. Breast Cancer Res.

[22] Wu Y, Zhang Y, Wang M, Li Q, Qu Z, Shi V, Kraft P, Kim S, Gao Y, Pak J, Youngster S, Horak ID, Greenberger LM (2013). Downregulation of HER3 by a novel antisense oligonucleotide, EZN-3920, improves the antitumor activity of EGFR and HER2 tyrosine kinase inhibitors in animal models. Mol Cancer Ther.

[23] Dufey E, Urra H, Hetz C (2015). ER proteostasis addiction in cancer biology: Novel concepts. Semin Cancer Biol.

[24] Liu Y, Ye Y (2011). Proteostasis regulation at the endoplasmic reticulum: a new perturbation site for targeted cancer therapy. Cell Res.

[25] Singh N, Joshi R, Komurov K (2015). HER2-mTOR signaling-driven breast cancer cells require ER-associated degradation to survive. Sci Signal.

[26] Milanezi F, Carvalho S, Schmitt FC (2008). EGFR/HER2 in breast cancer: a biological approach for molecular diagnosis and therapy. Expert Rev Mol Diagn.

[27] Jardines L, Weiss M, Fowble B, Greene M (1993). neu(c-erbB-2/HER2) and the epidermal growth factor receptor (EGFR) in breast cancer. Pathobiology.

[28] Martin V, Botta F, Zanellato E, Molinari F, Crippa S, Mazzucchelli L, Frattini M (2012). Molecular characterization of EGFR and EGFR-downstream pathways in triple negative breast carcinomas with basal like features. Histol Histopathol.

[29] Wang K, Ma Q, Ren Y, He J, Zhang Y, Zhang Y, Chen W (2007). Geldanamycin destabilizes HER2 tyrosine kinase and suppresses Wnt/beta-catenin signaling in HER2 overexpressing human breast cancer cells. Oncol Rep.

[30] Zheng FF, Kuduk SD, Chiosis G, Munster PN, Sepp-Lorenzino L, Danishefsky SJ, Rosen N (2000). Identification of a geldanamycin dimer that induces the selective degradation of HER-family tyrosine kinases. Cancer Res.

[31] Li YP, Chen JJ, Shen JJ, Cui J, Wu LZ, Wang Z, Li ZR (2015). Synthesis and biological evaluation of geldanamycin analogs against human cancer cells. Cancer Chemother Pharmacol.

[32] Xiong MP, Yanez JA, Remsberg CM, Ohgami Y, Kwon GS, Davies NM, Forrest ML (2008). Formulation of a geldanamycin prodrug in mPEG-b-PCL micelles greatly enhances tolerability and pharmacokinetics in rats. J Control Release.

[33] Ferreira RB, Law ME, Jahn SC, Davis BJ, Heldermon CD, Reinhard M, Castellano RK, Law BK (2015). Novel agents that downregulate EGFR, HER2, and HER3 in parallel. Oncotarget.

[34] Marciniak SJ, Yun CY, Oyadomari S, Novoa I, Zhang Y, Jungreis R, Nagata K, Harding HP, Ron D (2004). CHOP induces death by promoting protein synthesis and oxidation in the stressed endoplasmic reticulum. Genes Dev.

[35] Yamaguchi H, Wang HG (2004). CHOP is involved in endoplasmic reticulum stress-induced apoptosis by enhancing DR5 expression in human carcinoma cells. J Biol Chem.

[36] Li F, Guo Y, Sun S, Jiang X, Tang B, Wang Q, Wang L (2008). Free cholesterol-induced macrophage apoptosis is mediated by inositol-requiring enzyme 1 alpha-regulated activation of Jun N-terminal kinase. Acta Biochim Biophys Sin (Shanghai).

[37] Kumar R, Azam S, Sullivan JM, Owen C, Cavener DR, Zhang P, Ron D, Harding HP, Chen JJ, Han A, White BC, Krause GS, DeGracia DJ (2001). Brain ischemia and reperfusion activates the eukaryotic initiation factor 2alpha kinase, PERK. J Neurochem.

[38] Scheuner D, Song B, McEwen E, Liu C, Laybutt R, Gillespie P, Saunders T, Bonner-Weir S, Kaufman RJ (2001). Translational control is required for the unfolded protein response and *in vivo* glucose homeostasis. Mol Cell.

[39] Whittle JR, Lewis MT, Lindeman GJ, Visvader JE (2015). Patient-derived xenograft models of breast cancer and their predictive power. Breast Cancer Res.

[40] DeRose YS, Wang G, Lin YC, Bernard PS, Buys SS, Ebbert MT, Factor R, Matsen C, Milash BA, Nelson E, Neumayer L, Randall RL, Stijleman IJ (2011). Tumor grafts derived from women with breast cancer authentically reflect tumor pathology, growth, metastasis and disease outcomes. Nat Med.

[41] Palechor-Ceron N, Suprynowicz FA, Upadhyay G, Dakic A, Minas T, Simic V, Johnson M, Albanese C, Schlegel R, Liu X (2013). Radiation induces diffusible feeder cell factor(s) that cooperate with ROCK inhibitor to conditionally reprogram and immortalize epithelial cells. Am J Pathol.

[42] Liu X, Ory V, Chapman S, Yuan H, Albanese C, Kallakury B, Timofeeva OA, Nealon C, Dakic A, Simic V, Haddad BR, Rhim JS, Dritschilo A (2012). ROCK inhibitor and feeder cells induce the conditional reprogramming of epithelial cells. Am J Pathol.

[43] Chakrabarty A, Rexer BN, Wang SE, Cook RS, Engelman JA, Arteaga CL (2010). H1047R phosphatidylinositol 3-kinase mutant enhances HER2-mediated transformation by heregulin production and activation of HER3. Oncogene.

[44] Martin KA, Merenick BL, Ding M, Rzucidlo EM, Kozul CD, Brown DJ, Chiu HY, Shyu M, Drapeau BL, Wagner RJ, Powell RJ (2007). Rapamycin promotes vascular smooth muscle cell differentiation through insulin receptor substrate-1/phosphatidylinositol 3-kinase/Akt2 feedback signaling. J Biol Chem.

[45] O’Reilly KE, Rojo F, She QB, Solit D, Mills GB, Smith D, Lane H, Hofmann F, Hicklin DJ, Ludwig DL, Baselga J, Rosen N (2006). mTOR inhibition induces upstream receptor tyrosine kinase signaling and activates Akt. Cancer Res.

[46] Sadowski K, Kotulska K, Jozwiak S (2016). Management of side effects of mTOR inhibitors in tuberous sclerosis patients. Pharmacol Rep.

[47] Sampson JR (2009). Therapeutic targeting of mTOR in tuberous sclerosis. Biochem Soc Trans.

[48] Bai F, Ho Lim C, Jia J, Santostefano K, Simmons C, Kasahara H, Wu W, Terada N, Jin S (2015). Directed Differentiation of Embryonic Stem Cells Into Cardiomyocytes by Bacterial Injection of Defined Transcription Factors. Sci Rep.

[49] Singh AM, Li FQ, Hamazaki T, Kasahara H, Takemaru K, Terada N (2007). Chibby, an antagonist of the Wnt/beta-catenin pathway, facilitates cardiomyocyte differentiation of murine embryonic stem cells. Circulation.

[50] Farnie G, Willan PM, Clarke RB, Bundred NJ (2013). Combined inhibition of ErbB1/2 and Notch receptors effectively targets breast ductal carcinoma *in situ* (DCIS) stem/progenitor cell activity regardless of ErbB2 status. PLoS One.

[51] Kaji EH, Lodish HF (1993). *In vitro* unfolding of retinol-binding protein by dithiothreitol. Endoplasmic reticulum-associated factors. J Biol Chem.

[52] Braakman I, Helenius J, Helenius A (1992). Manipulating disulfide bond formation and protein folding in the endoplasmic reticulum. EMBO J.

[53] Workman P (2002). Cancer genome targets: RAF-ing up tumor cells to overcome oncogene addiction. Expert Rev Anticancer Ther.

[54] Kaelin WG (2005). The concept of synthetic lethality in the context of anticancer therapy. Nat Rev Cancer.

[55] Garber K (2002). Synthetic lethality: killing cancer with cancer. J Natl Cancer Inst.

[56] Livasy CA, Karaca G, Nanda R, Tretiakova MS, Olopade OI, Moore DT, Perou CM (2006). Phenotypic evaluation of the basal-like subtype of invasive breast carcinoma. Mod Pathol.

[57] Monaghan P, Clarke CL, Perusinghe NP, Ormerod MG, O’Hare MJ (1995). Epidermal growth factor receptor expression on human breast luminal and basal cells *in vitro*. Epithelial Cell Biol.

[58] Arteaga CL (2007). HER3 and mutant EGFR meet MET. Nat Med.

[59] Zhang Z, Wang J, Ji D, Wang C, Liu R, Wu Z, Liu L, Zhu D, Chang J, Geng R, Xiong L, Fang Q, Li J (2014). Functional genetic approach identifies MET, HER3, IGF1R, INSR pathways as determinants of lapatinib unresponsiveness in HER2-positive gastric cancer. Clin Cancer Res.

[60] Jia Y, Zhang Y, Qiao C, Liu G, Zhao Q, Zhou T, Chen G, Li Y, Feng J, Li Y, Zhang Q, Peng H (2013). IGF-1R and ErbB3/HER3 contribute to enhanced proliferation and carcinogenesis in trastuzumab-resistant ovarian cancer model. Biochem Biophys Res Commun.

[61] Desbois-Mouthon C, Baron A, Blivet-Van Eggelpoel MJ, Fartoux L, Venot C, Bladt F, Housset C, Rosmorduc O (2009). Insulin-like growth factor-1 receptor inhibition induces a resistance mechanism via the epidermal growth factor receptor/HER3/AKT signaling pathway: rational basis for cotargeting insulin-like growth factor-1 receptor and epidermal growth factor receptor in hepatocellular carcinoma. Clin Cancer Res.

[62] Wada R, Yagihashi S, Naito Z (2016). mRNA expression of delta-HER2 and its clinicopathological correlation in HER2-overexpressing breast cancer. Mol Med Rep.

[63] Alajati A, Sausgruber N, Aceto N, Duss S, Sarret S, Voshol H, Bonenfant D, Bentires-Alj M (2013). Mammary tumor formation and metastasis evoked by a HER2 splice variant. Cancer Res.

[64] Cittelly DM, Das PM, Salvo VA, Fonseca JP, Burow ME, Jones FE (2010). Oncogenic HER2{Delta}16 suppresses miR-15a/16 and deregulates BCL-2 to promote endocrine resistance of breast tumors. Carcinogenesis.

[65] Yuan H, Myers S, Wang J, Zhou D, Woo JA, Kallakury B, Ju A, Bazylewicz M, Carter YM, Albanese C, Grant N, Shad A, Dritschilo A (2012). Use of reprogrammed cells to identify therapy for respiratory papillomatosis. N Engl J Med.

[66] Law ME, Corsino PE, Jahn SC, Davis BJ, Chen S, Patel B, Pham K, Lu J, Sheppard B, Norgaard P, Hong J, Higgins P, Kim JS (2013). Glucocorticoids and histone deacetylase inhibitors cooperate to block the invasiveness of basal-like breast cancer cells through novel mechanisms. Oncogene.

[67] Law ME, Ferreira RB, Davis BJ, Higgins PJ, Kim JS, Castellano RK, Chen S, Luesch H, Law BK (2016). CUB domain-containing protein 1 and the epidermal growth factor receptor cooperate to induce cell detachment. Breast Cancer Res.

[68] Greulich H, Chen TH, Feng W, Janne PA, Alvarez JV, Zappaterra M, Bulmer SE, Frank DA, Hahn WC, Sellers WR, Meyerson M (2005). Oncogenic transformation by inhibitor-sensitive and -resistant EGFR mutants. PLoS Med.

[69] Greulich H, Kaplan B, Mertins P, Chen TH, Tanaka KE, Yun CH, Zhang X, Lee SH, Cho J, Ambrogio L, Liao R, Imielinski M, Banerji S (2012). Functional analysis of receptor tyrosine kinase mutations in lung cancer identifies oncogenic extracellular domain mutations of ERBB2. Proc Natl Acad Sci U S A.

[70] Law BK, Chytil A, Dumont N, Hamilton EG, Waltner-Law ME, Aakre ME, Covington C, Moses HL (2002). Rapamycin potentiates transforming growth factor beta-induced growth arrest in nontransformed, oncogene-transformed, and human cancer cells. Mol Cell Biol.

[71] Wang Y, Shen J, Arenzana N, Tirasophon W, Kaufman RJ, Prywes R (2000). Activation of ATF6 and an ATF6 DNA binding site by the endoplasmic reticulum stress response. J Biol Chem.

[72] Chen X, Shen J, Prywes R (2002). The luminal domain of ATF6 senses endoplasmic reticulum (ER) stress and causes translocation of ATF6 from the ER to the Golgi. J Biol Chem.

[73] Law BK, Norgaard P, Gnudi L, Kahn BB, Poulson HS, Moses HL (1999). Inhibition of DNA synthesis by a farnesyltransferase inhibitor involves inhibition of the p70(s6k) pathway. J Biol Chem.

