# Supplementary material for: Disulfide bond disrupting agents activate the unfolded protein response in EGFR- and HER2-positive breast tumor cells
Source: Oncotarget. 2017 Mar 7;8(17):28971–89. doi: 10.18632/oncotarget.15952 (PMC5438706; doi:10.18632/oncotarget.15952)
Supplement: Supplementary file 1 [file oncotarget-08-28971-s001.pdf]

Disulfide bond disrupting agents activate the unfolded protein response in EGFR- and HER2-positive breast tumor cells

SUPPLEMENTARY DATA

Synthesis of DDAs

General Methods: Reagents and solvents were purchased from commercial sources and used without further purification unless otherwise specified. ^{1}H and ^{13}C NMR spectra were recorded using commercially obtained (Cambridge Isotope Laboratories) deuterated solvents on a Varian Inova-500 (^{1}H at 500 MHz; ^{13}C at 126 MHz) spectrometer. Chemical shifts (δ) are given in parts per million (ppm) relative to tetramethylsilane (TMS) and referenced to residual protonated solvent ($CDCl_3$: δ H 7.26 ppm, δ C 77.23 ppm; CD_3OD: δ H 4.87 ppm, δ C 49.00 ppm; DMSO-d_6: δ H 2.50 ppm, δ C 39.52 ppm; D_2O: δ H 4.79 ppm). Coupling constants are given in Hz. Spin multiplicities are presented by the following symbols: s (singlet), bs (broad singlet), d (doublet), t (triplet), q (quartet), p (pentet), and m (multiplet). Electrospray ionization (ESI) or Direct Analysis in Real Time (DART) high resolution mass spectra (HRMS) were recorded on an Agilent 6200 ESI-TOF instrument, operating in positive or negative ion mode as stated, with methanol as the carrier solvent for ESI experiments. Matrix-Assisted Laser Desorption Ionization (MALDI) HRMS were recorded on a Bruker Microflex LRF MALDI TOF instrument, with a matrix mixture of DHB/analyte 1:1.

RBF3 and DTDO were synthesized as described in our previous publication [1]. NMR spectra of the synthesized compounds are shown in Supplementary Figure 2.

A. Bn-DDA: To a stirring solution of benzene-1,3,5-triyltrimethanethiol (150 mg, 0.693 mmol) and DTDO (475 mg, 3.12 mmol) in MeOH/THF 2:1 (6.3 mL) at 0 °C, a solution of NaOMe (freshly prepared from 48 mg of Na^0 and 1.5 mL of MeOH) was added dropwise over 15 min. The reaction was allowed to stir for an additional 15 min at 0 °C and acetone was added to form a precipitate, which was subsequently collected by filtration and washed with acetone. The crude material was redissolved in a minimal amount of MeOH and acetone was added until turbidity was apparent. The solution was centrifuged, the supernatant was collected, and more acetone was added to induce precipitation. The resulting solid was then collected by filtration, washed with acetone, and dried under vacuum to afford the product (391 mg, 0.529 mmol, 76% yield) as a white solid. ^{1}H NMR (D_2O, 500 MHz): δ 7.23 (s, 3H), 3.86 (s, 6H), 2.57 (m, 6H), 2.35 (m, 6H), 1.71 (m, 6H), 1.62 (m, 6H). ^{13}C NMR (D_2O, 126 MHz): δ 138.23, 129.46, 60.67, 42.34, 37.75, 28.23, 21.32; HRMS-MALDI: *m/z* [M+H]+ calcd for $[C_{21}H_{34}Na_3O_6S_9]^+$: 738.9529; found: 738.9534.

B. PEMP-DDA: To a stirring solution of pentaerythritol tetrakis(3-mercaptopropionate) (382 µL, 1.00 mmol) and DTDO (670 mg, 4.40 mmol) in EtOH/THF 3:1 (12 mL) at 0 °C, a solution of NaOMe (freshly prepared from 92 mg of Na^0 and 3 mL of MeOH) was added dropwise over 15 min. The reaction was allowed to stir for an additional 15 min at 0 °C, after which acetone was added to form a precipitate. The precipitate was collected by filtration, washed with acetone, and then redissolved in a minimal amount of MeOH. Acetone was added until turbidity was apparent, the solution was centrifuged, and the supernatant was collected and more acetone was added to induce precipitation. The resulting solid was then collected by filtration, washed with acetone, and dried under vacuum to afford the product (944 mg, 0.796 mmol, 80% yield) as a white solid. ^{1}H NMR (D_2O, 500 MHz): δ 4.18 (s, 8H), 2.89 (m, 8H), 2.79 (m, 8H), 2.67 (m, 8H), 2.27 (m, 8H), 1.69 (m, 8H), 1.56 (m, 8H); ^{13}C NMR (D_2O, 126 MHz): δ 172.96, 62.92, 60.58, 41.94, 37.81, 33.86, 32.64, 28.22, 21.22; HRMS-ESI: *m/z* [M–Na]$^-$ calcd for $[C_{33}H_{56}Na_3O_{16}S_{12}]^-$: 1160.9915; found: 1160.9946.

cis- and trans-DAcDTDO were prepared from dithioerythritol (DTE) and dithiothreitol (DTT) using similar procedures to those described by Field and Khim [2].

C. cis-DAcDTDO: ^{1}H NMR (DMSO-d_6, 500 MHz): δ 5.43 – 5.40 (m, 1H), 5.34 (dt, J = 10.9, 3.1 Hz, 1H), 3.98 (dd, J = 13.1, 3.4 Hz, 1H), 3.78 (dd, J = 13.0, 11.0 Hz, 1H), 3.70 (dd, J = 15.1, 5.6 Hz, 1H), 3.65 – 3.58 (m, 1H), 2.12 (s, 3H), 2.02 (s, 3H); ^{13}C NMR (DMSO-d_6, 126 MHz): δ 169.93, 169.35, 69.47, 64.34, 58.87, 34.77, 21.11, 21.00; HRMS-ESI: *m/z* [M+Na]$^+$ calcd for $[C_8H_{12}NaO_6S_2]^+$: 290.9968; found: 290.9972.

D. trans-DAcDTDO: ^{1}H NMR ($CDCl_3$, 500 MHz): δ 5.44 (td, J = 8.3, 3.8 Hz, 1H), 5.13 (td, J = 8.3, 3.1 Hz, 1H), 3.81 (dd, J = 13.6, 3.8 Hz, 1H), 3.66 (dd, J = 13.6, 8.7 Hz, 1H), 3.61 – 3.53 (m, 1H), 3.45 (dd, J = 14.6, 8.7 Hz, 1H), 2.13 (s, 3H), 2.11 (s, 3H); ^{13}C NMR ($CDCl_3$, 126 MHz): δ 169.67, 169.15, 69.40, 68.10, 60.74, 33.04,

20.89, 20.78; HRMS-DART: *m/z* $[M+NH_4]^+$ calcd for $[C_8H_{16}NO_6S_2]^+$: 286.0414; found: 286.0428.

cis- and trans-DHDTDO were prepared by a procedure adapted from literature [3]: To DAcDTDO (1.00 g, 3.73 mmol) was added 7 M NH_3 in MeOH (100 mL) dropwise over 15 min under argon at rt. The solid went into solution as the NH_3 solution was added and the solution turned yellow. After 1.5 h, the reaction mixture was concentrated under vacuum, and the crude oil was purified by column chromatography (SiO_2, 5% MeOH in CH_2Cl_2) to give the DHDTDO as a white solid.

E. cis-DHDTDO: 0.560 g (3.04 mmol, 81% yield). ^1H NMR (CD_3OD, 500 MHz): *d* 4.23 – 4.18 (m, 1H), 4.13 (dt, *J* = 11.0, 2.9 Hz, 1H), 3.66 (dd, *J* = 12.7, 11.0 Hz, 1H), 3.47 (dd, *J* = 14.7, 1.2 Hz, 1H), 3.42 – 3.33 (m, 2H); ^{13}C NMR (CD_3OD, 126 MHz): δ 71.45, 65.93, 61.63, 38.65; HRMS-ESI: *m/z* $[M+Na]^+$ calcd for $[C_4H_8NaO_4S_2]^+$: 206.9756; found: 206.9755.

F. trans-DHDTDO: 0.520 g (2.83 mmol, 76% yield). ^1H NMR (CD_3OD, 500 MHz): δ 3.97 (ddd, *J* = 10.3, 8.6, 4.0 Hz, 1H), 3.79 – 3.68 (m, 2H), 3.51 (dd, *J* = 13.2, 10.4 Hz, 1H), 3.37 – 3.29 (m, 1H), 3.22 (dd, *J* = 14.2, 10.2 Hz, 1H); ^{13}C NMR (CD_3OD, 126 MHz): δ 77.83, 77.36, 65.43, 36.21; HRMS-ESI: *m/z* $[M+Na]^+$ calcd for $[C_4H_8NaO_4S_2]^+$: 206.9756; found: 206.9760.

REFERENCES

1. Ferreira RB, Law ME, Jahn SC, Davis BJ, Heldermon CD, Reinhard M, et al. Novel agents that downregulate EGFR, HER2, and HER3 in parallel. Oncotarget. 2015 Apr 30;6:10445-59.
2. Field L, Khim YH. Organic disulfides and related substances. 33. Sodium 4-(2-acetamidoethyldithio) butanesulfinate and related compounds as antiradiation drugs. J Med Chem. 1972 Mar;15:312-5.
3. Mayasundari A, Rice WG, Diminnie JB, Baker DC. Synthesis, resolution, and determination of the absolute configuration of the enantiomers of cis-4,5-dihydroxy-1,2-dithiane 1,1-dioxide, an HIV-1NCp7 inhibitor. Bioorg Med Chem. 2003 Jul 17;11:3215-9.

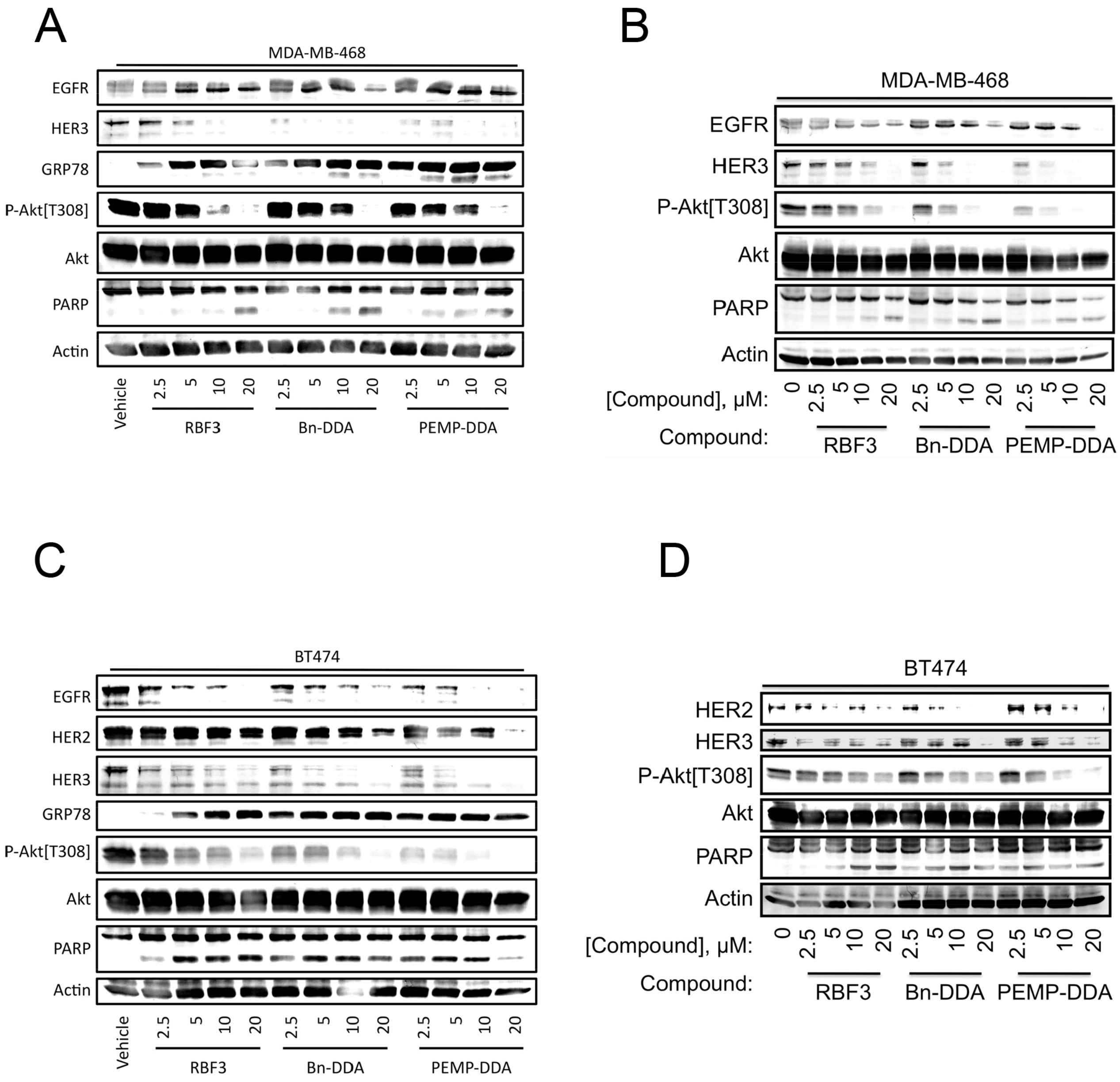

Supplementary Figure 1: Replicates of experiments with MDA-MB-468 (panels A and B) and BT474 (panels C and D) cells corresponding to Figures 5B and 6A, respectively. The experiments were carried out as described in the Figure 5 and Figure 6 legends.

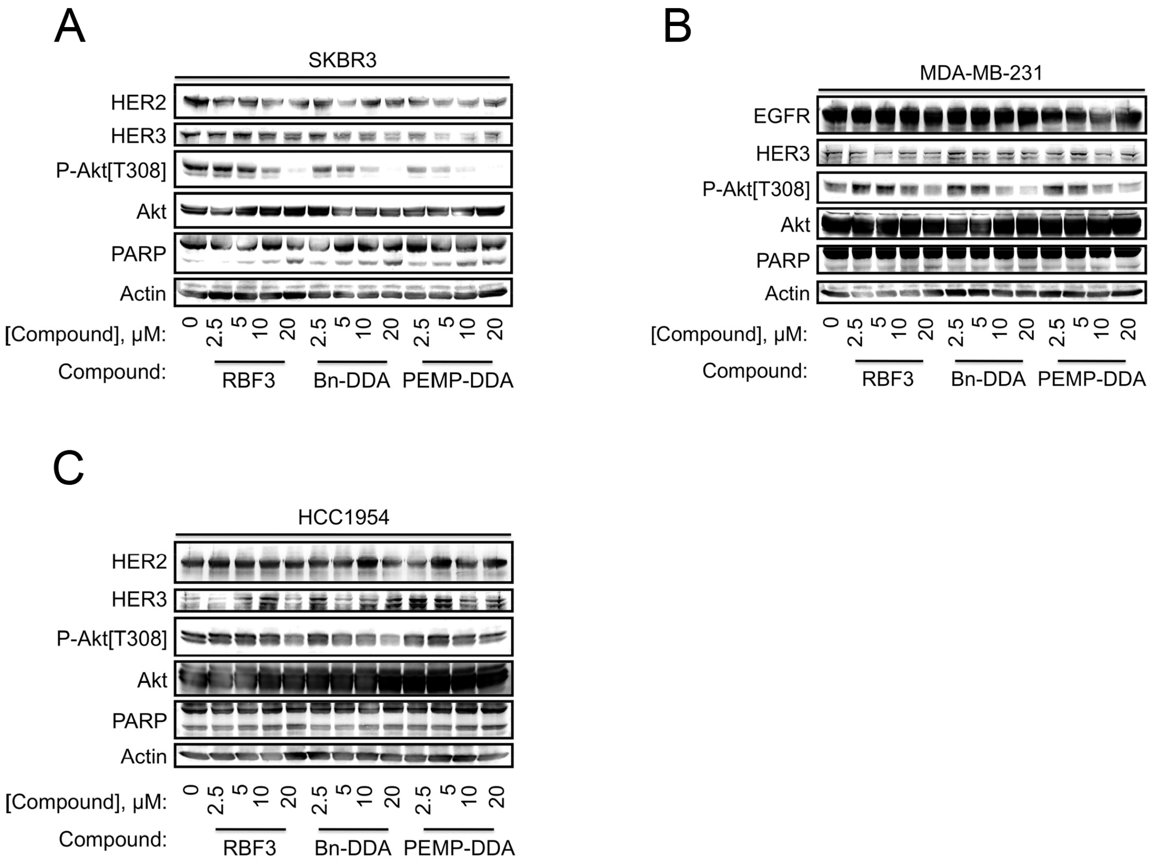

Supplementary Figure 2: Immunoblot analysis of samples from SKBR3 (A), MDA-MB-231 (B), and HCC1954 (C) cells treated as in Supplementary Figure 1 and Figures 5B and 6A.

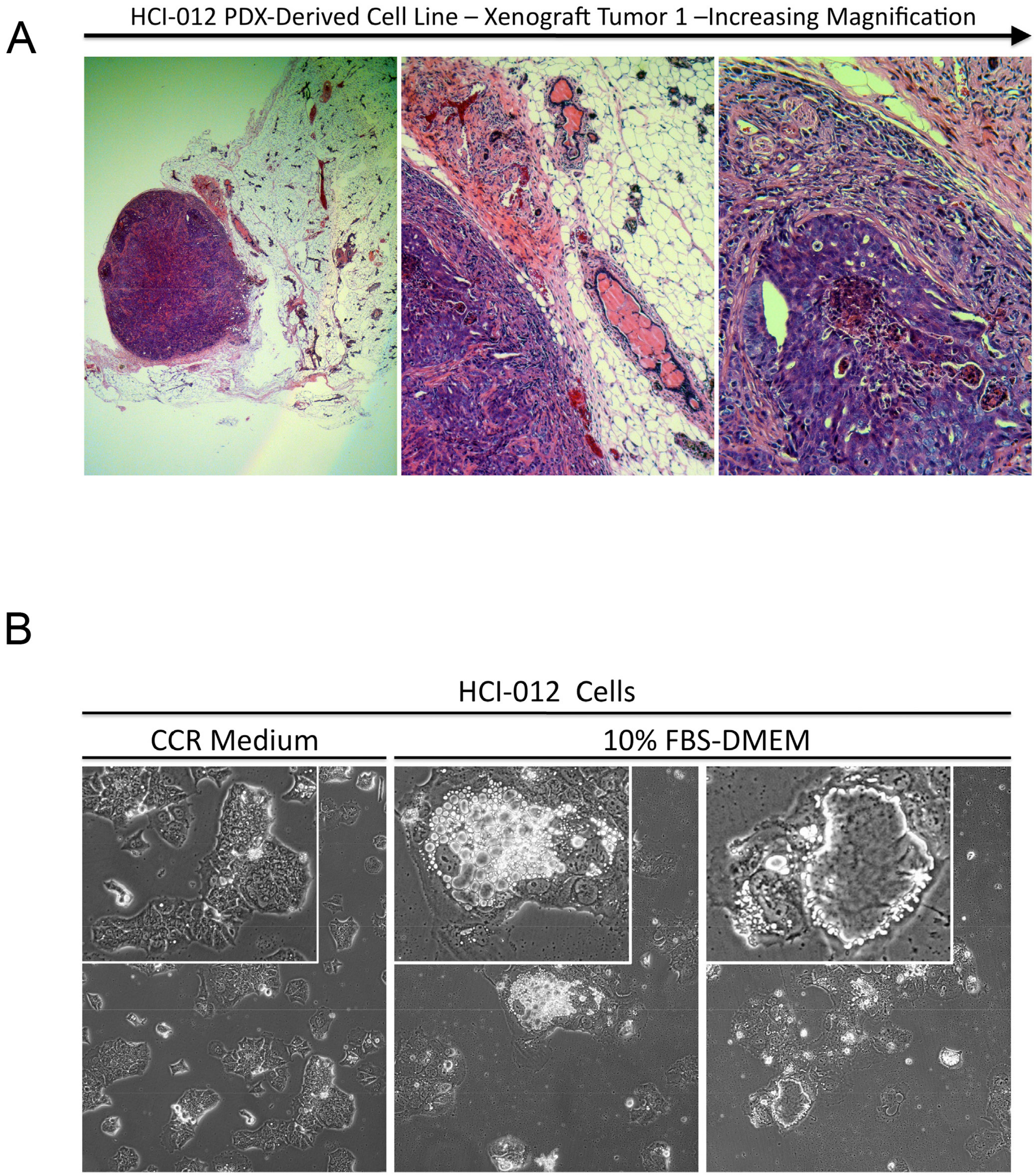

Supplementary Figure 3: Characterization of the HCI-012 PDX-derived cell line. **(A)** Micrograph of a Hematoxylin and Eosin (H&E)-stained section of a xenograft tumor derived from the HCI-012 cell line growing in a mouse mammary fat pad. **(B)** HCI-012 cells growing in Conditional Cell Reprogramming (CCR) Medium (left panel) or incubated in 10% FBS-DMEM (center and right panels). Note that when not grown in CCR medium the HCI-012 cells begin to senesce and die.

A. Bn-DDA:

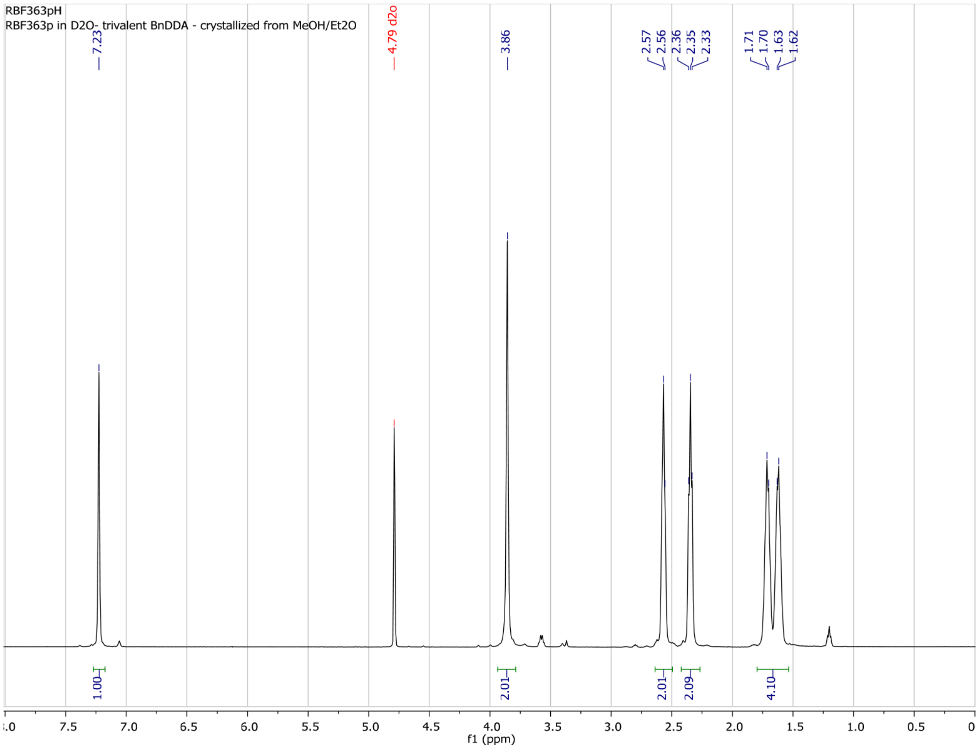

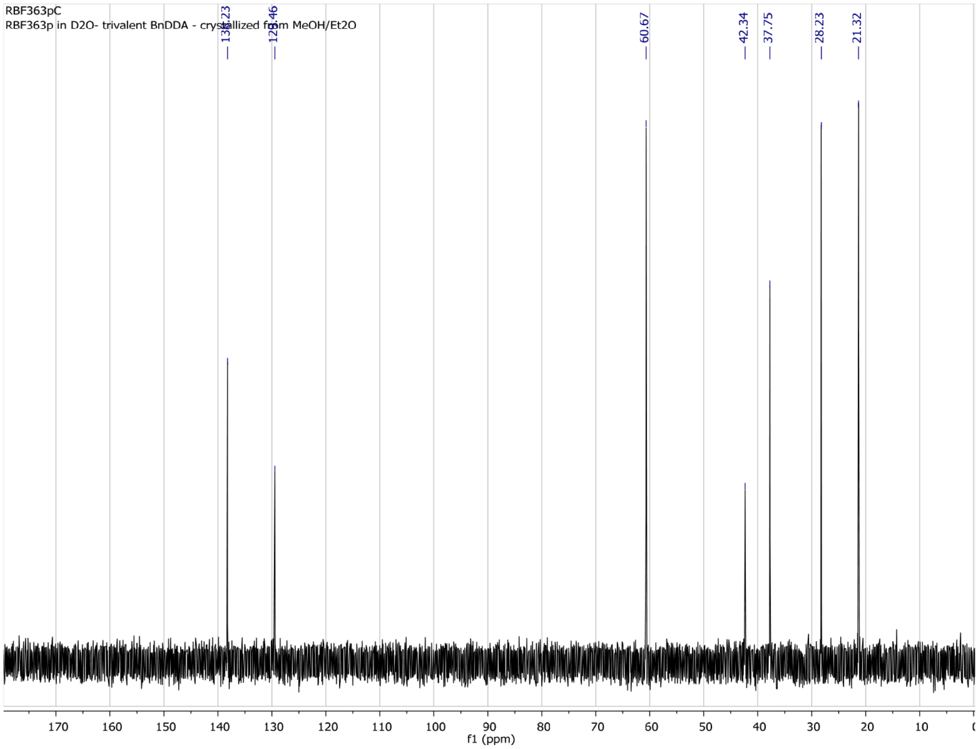

(*Continued*)

B. PEMP-DDA:

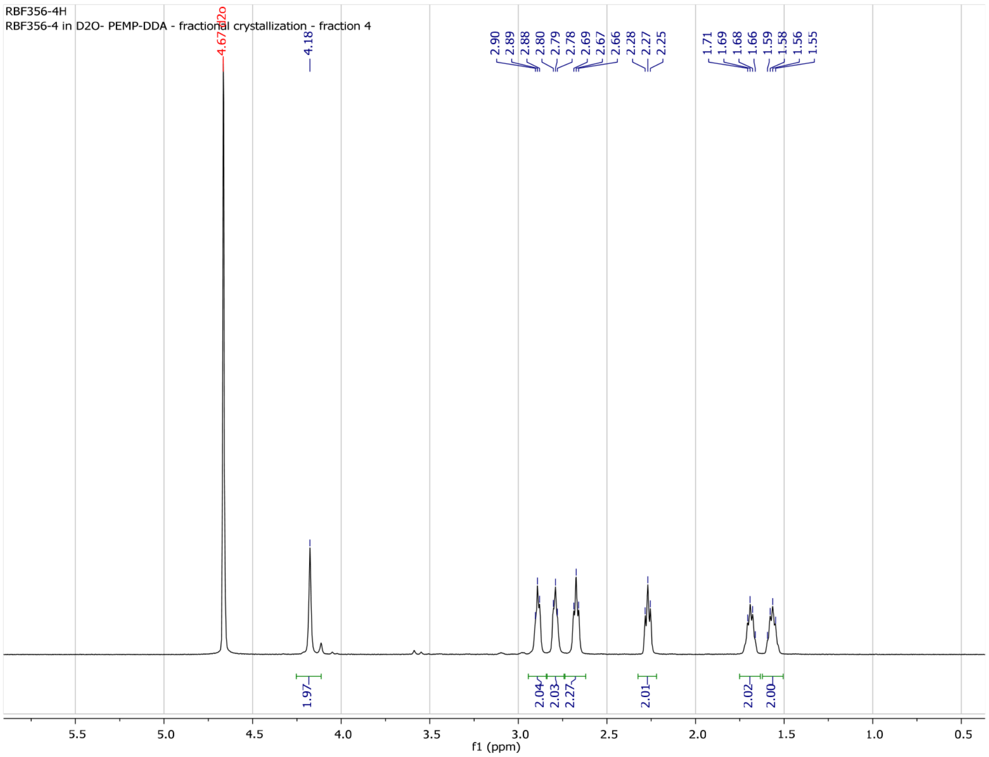

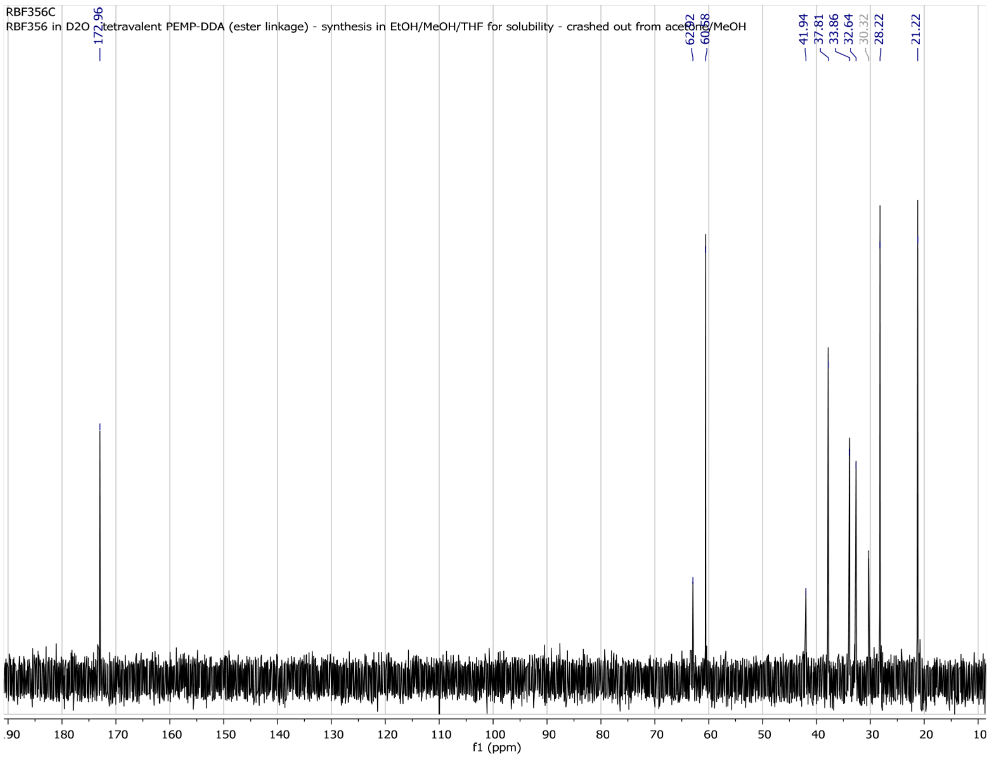

(*Continued*)

C. cis-DAcDTDO:

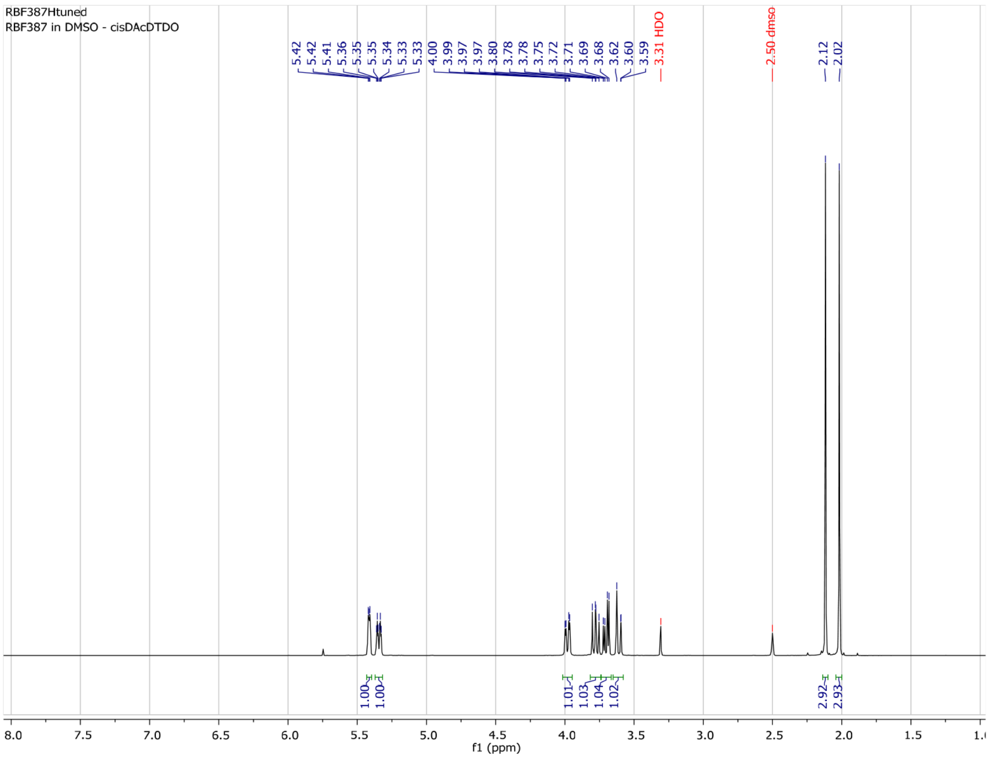

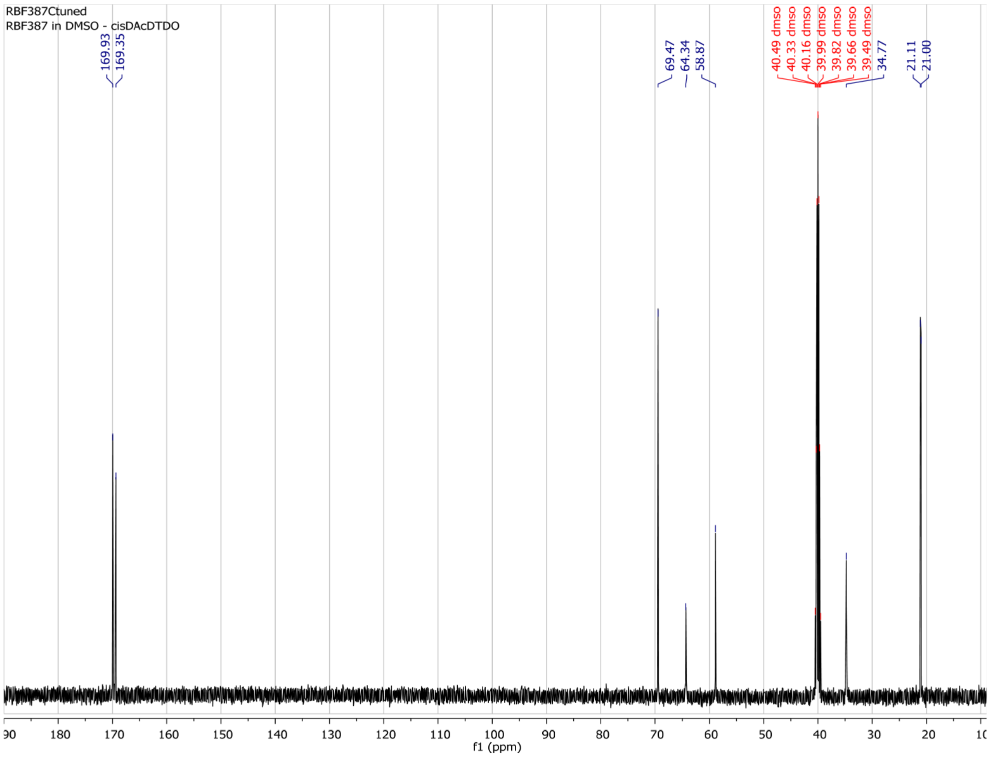

(*Continued*)

D. trans-DAcDTDO:

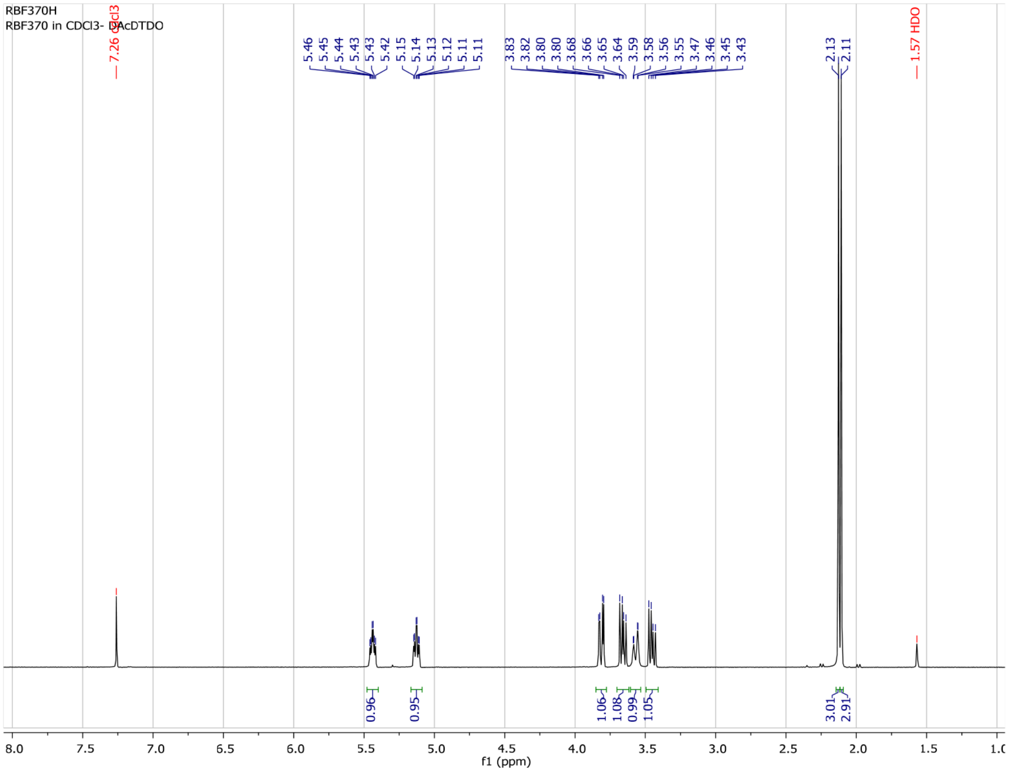

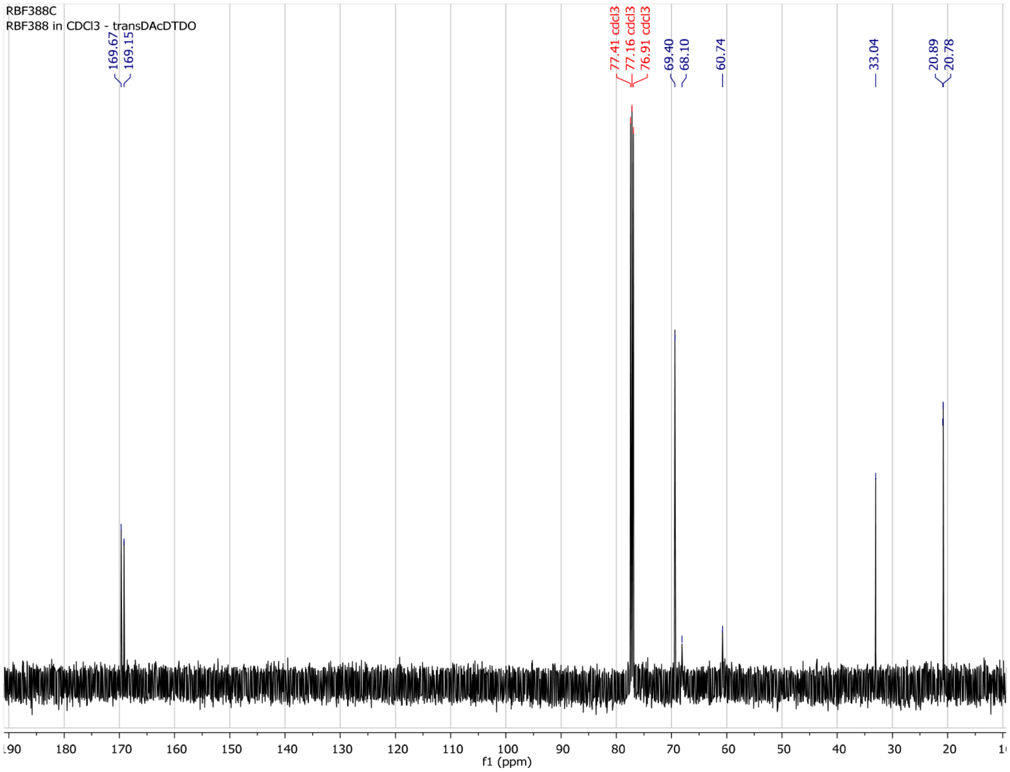

(*Continued*)

E. cis-DHDTDO:

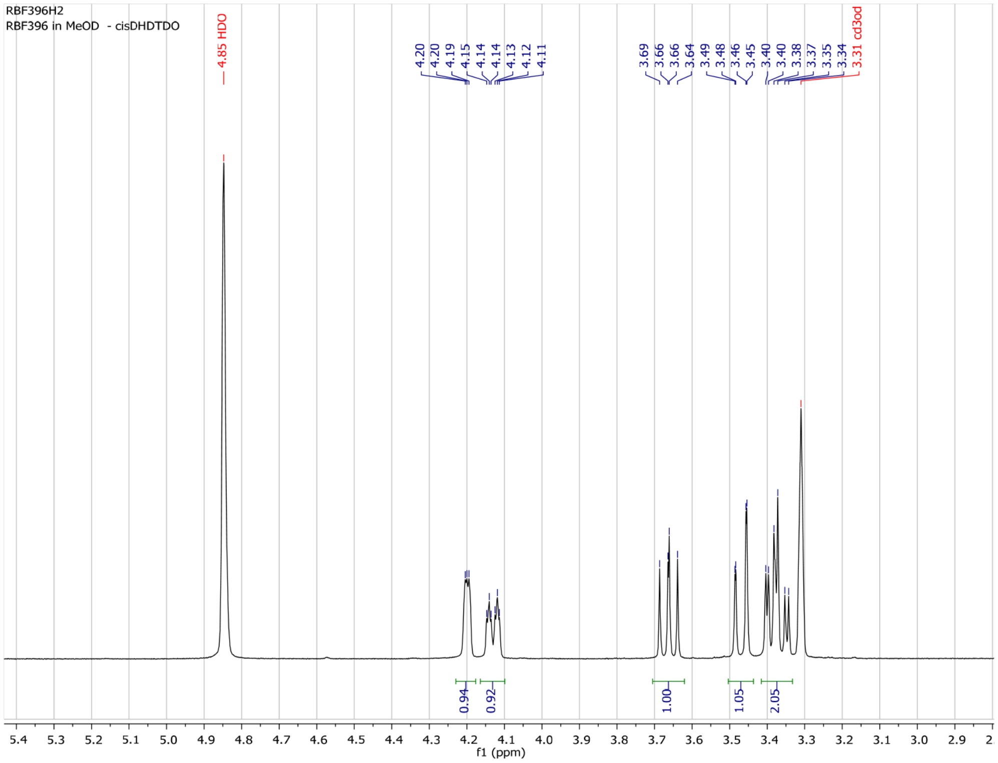

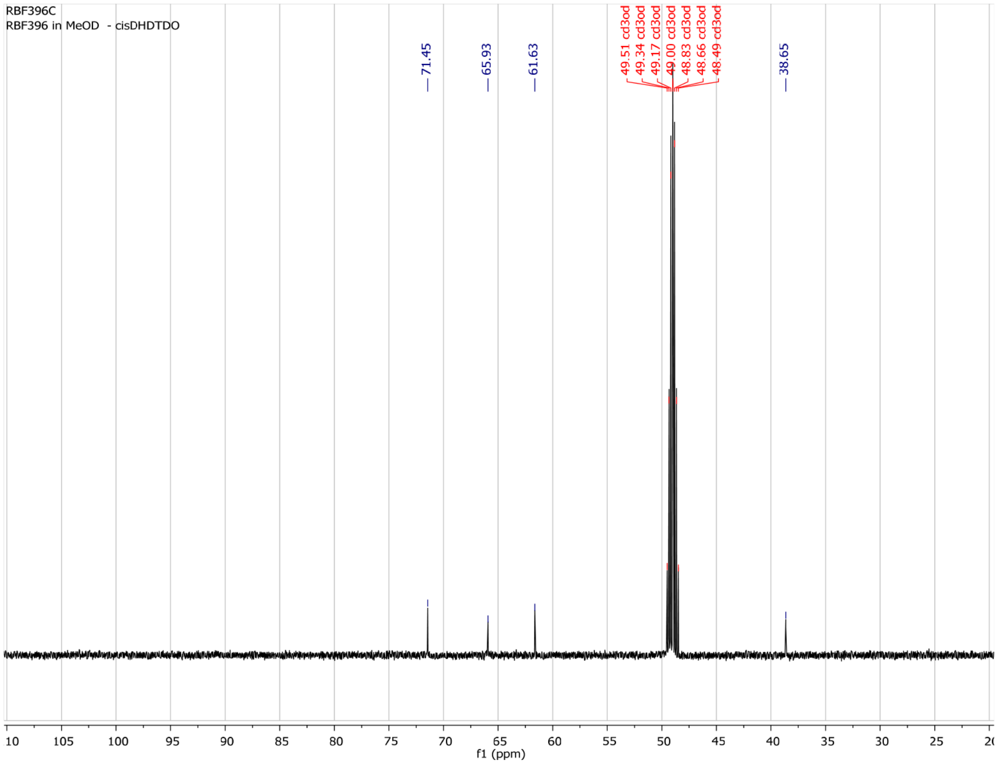

(Continued)

F. trans-DHDTDO:

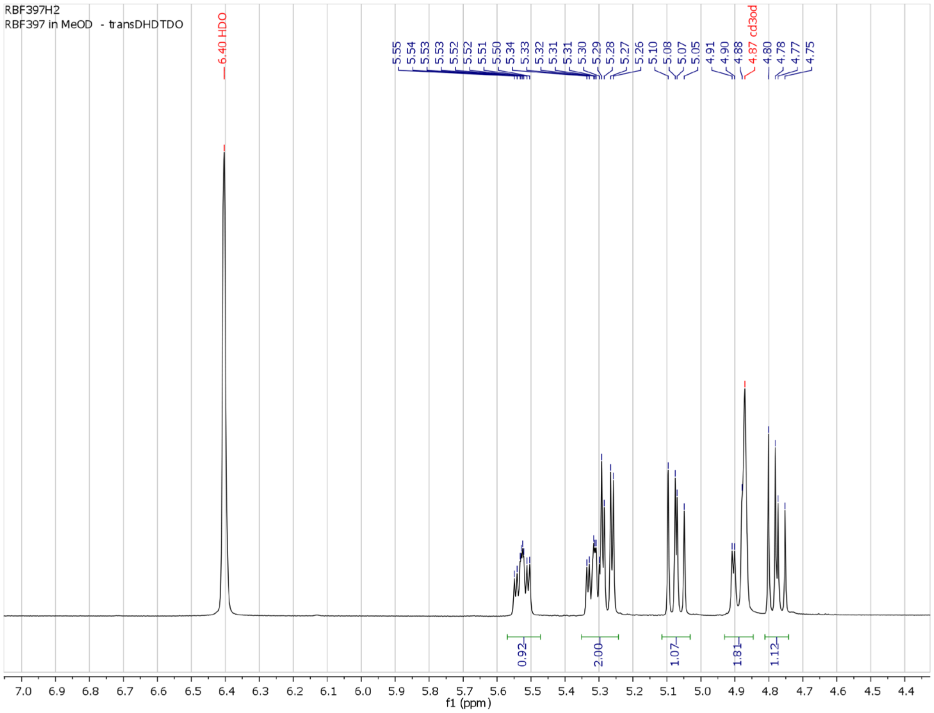

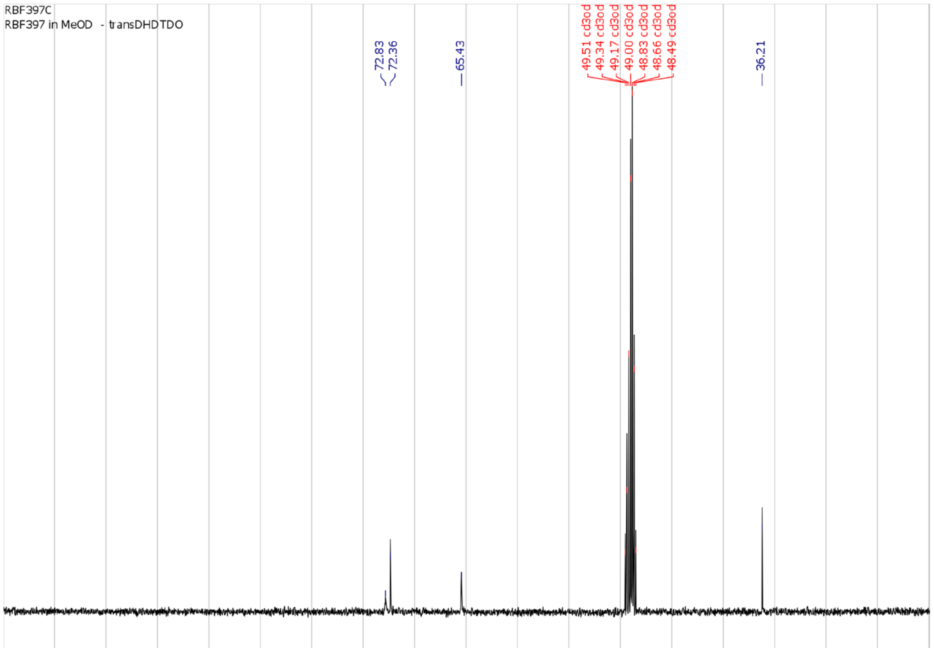

Supplementary Figure 4: NMR spectra of synthesized DDA compounds. **(A)** Bn-DDA, **(B)** PEMP-DDA, **(C)** cis-DAcDTDO, **(D)** trans-DAcDTDO, **(E)** cis-DHDTDO, **(F)** trans-DHDTO.

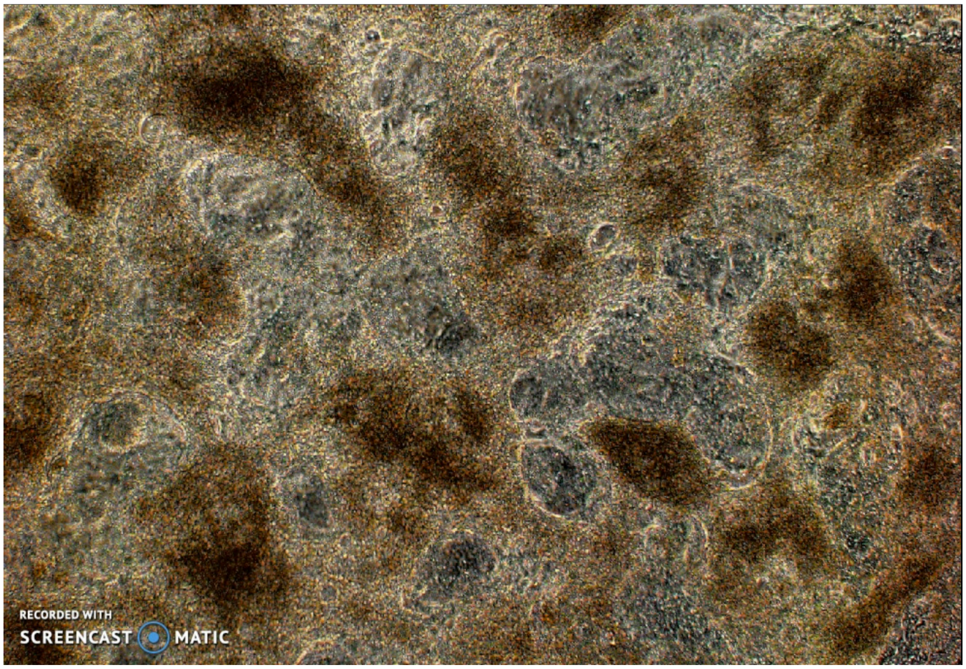

Supplementary Video 1: Video of beating cardiomyocytes derived from iPSCs captured after a 24 hour treatment with the vehicle control.

See Supplementary Video 1

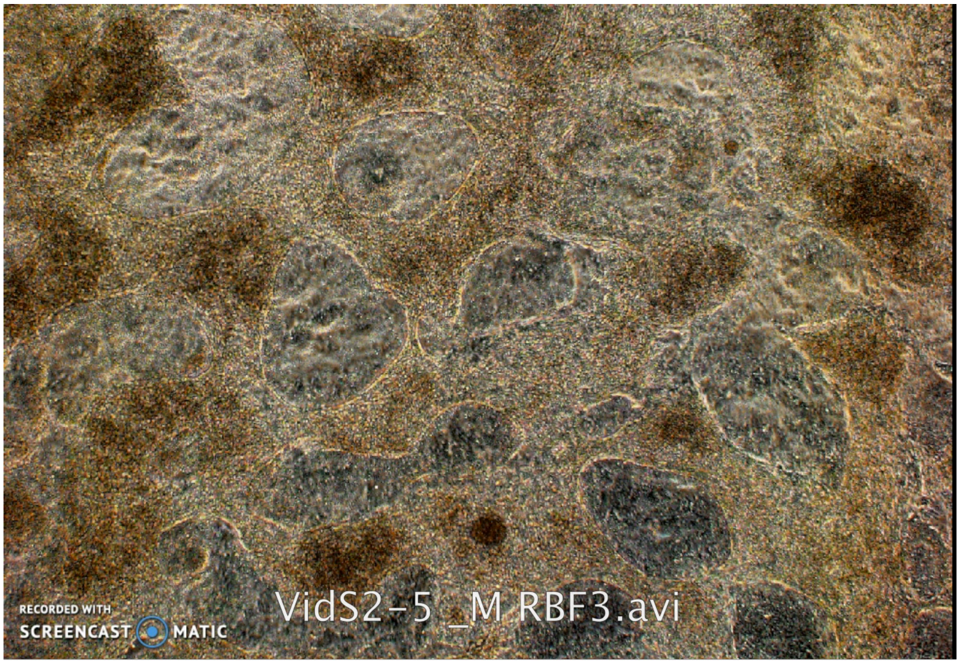

Supplementary Video 2: Video of beating cardiomyocytes derived from iPSCs captured after a 24 hour treatment with 5 µM RBF3.

See Supplementary Video 2

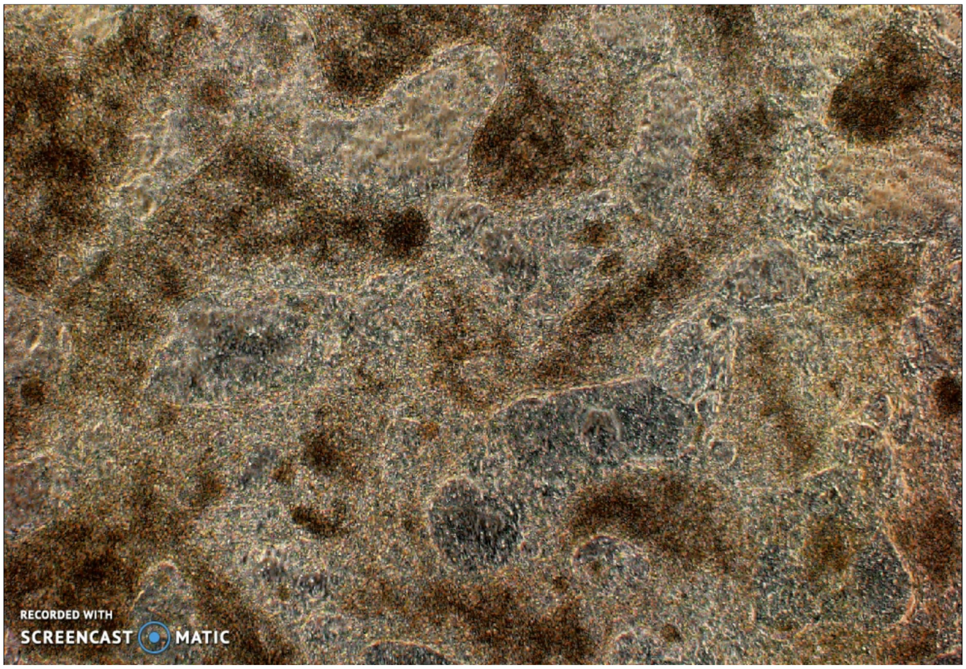

Supplementary Video 3: Video of beating cardiomyocytes derived from iPSCs captured after a 24 hour treatment with 20 µM RBF3.

See Supplementary Video 3
